# Truncated CD19 as a selection marker for the isolation of stem cell-derived β-cells

**DOI:** 10.1242/dmm.052376

**Published:** 2026-01-05

**Authors:** Luo Ting Huang, Raymond Jun-rui Gao, Dahai Zhang, Cuilan Nian, Willem Martzke, A. M. James Shapiro, Tatsuya Kin, Yaser Tahamtani, Francis C. Lynn

**Affiliations:** ^1^ BC Children's Hospital Research Institute, University of British Columbia, Vancouver, BC V5Z 4H4, Canada; ^2^ Department of Surgery, University of British Columbia, Vancouver, BC V5Z 1M9, Canada; ^3^ Clinical Islet Laboratory, University of Alberta Hospital, Edmonton, AB T6G 2B7, Canada; ^4^ School of Biomedical Engineering, University of British Columbia, Vancouver, BC V6T 1Z3, Canada

**Keywords:** CD19, Insulin, Stem cell-derived beta cells, Diabetes, Genome editing, Magnetic-activated cell sorting

## Abstract

Stem cell-derived β-cells (SCβ-cell) are a renewable and scalable alternative to cadaveric islets as a cell-replacement therapy for type 1 diabetes (T1D). However, heterogeneity within SCβ-cell cultures remains problematic for graft safety and function. Magnetic selection of SCβ-cells expressing a unique cell-surface marker may help deplete undesirable cell types and facilitate functional maturation. Here, we explored the transmembrane glycoprotein CD19 as a potential cell-surface marker for the enrichment of insulin-expressing SCβ-cells. Using CRISPR/Cas9 technology, we created a knock-in add-on of CD19-mScarlet downstream of insulin (INS) coding sequence exon 2 in human embryonic stem cells (hESCs). We developed and optimized a magnetic-activated cell sorting protocol for CD19-mScarlet-expressing cells, forming enriched SCβ-cell clusters with improved glucose-stimulated C-peptide secretion. This strategy holds promise to facilitate large-scale production of functional SCβ-cells for disease modeling and cell-replacement therapy.

## Teaser

**Summary:** We use genetic engineering of human embryonic stem cells to facilitate magnetic-activated cell sorting enrichment of insulin-producing stem cell-derived β cells.

## INTRODUCTION

Pancreatic β-cells maintain metabolic homeostasis by producing, storing and releasing insulin in response to fluctuations in blood glucose concentrations. Destruction or dysfunction of pancreatic β-cells leads to insufficient insulin production, chronic hyperglycemia and the development of diabetes. While traditional interventions, such as exogenous insulin administration, can restore normoglycemia, cadaveric islet transplantation remains one of the most effective treatment options for type 1 diabetes (T1D) (Shapiro, 2012; Shapiro et al., 2017). However, this procedure is limited by the scarce supply and inconsistent quality of islet donors, which poses a barrier to broader applications in a clinical setting (Bellin et al., 2012; Sneddon et al., 2018). Recently, human pluripotent stem cell-derived β-cells (SCβ-cells) have emerged as a renewable, scalable and, thus, promising alternative for cadaveric pancreatic islet transplantation (Shapiro and Verhoeff, 2023). Over the past decades, several multi-stage differentiation protocols have recapitulated the embryogenesis of pancreatic β-cells from definitive endoderm to pancreatic progenitors to endocrine progenitors and pancreatic islets (D'Amour et al., 2006; Hogrebe et al., 2020; Korytnikov and Nostro, 2016; Kroon et al., 2008; Pagliuca et al., 2014; Rezania et al., 2014). Recent efforts have focused on generating mature, insulin-expressing (INS+) pancreatic β-cells that secrete insulin in response to glucose stimulation (Balboa et al., 2022; Nair et al., 2019; Russ et al., 2015; Velazco-Cruz et al., 2019). Heterogeneity within SCβ-cell cultures remains problematic, as undesired populations of polyhormonal cells and proliferating pancreatic progenitors may interfere with graft safety and function (Hiyoshi et al., 2022; Robert et al., 2018; Veres et al., 2019).

Enzymatic dissociation and controlled reaggregation have been used to enrich for pancreatic endocrine cells and facilitate maturation (Agulnick et al., 2015; Kelly et al., 2011; Velazco-Cruz et al., 2019). Designing and utilizing genetically engineered human pluripotent stem cells (hPSCs) for the final enrichment of SC-derived β-cells is an approach that has been studied recently. Notably, Nair et al. (2019) generated glucose-responsive, enhanced β-clusters by sorting for insulin-expressing cells at the immature β-cell stage. Their method relies on fluorescence-activated cell sorting (FACS) of a transgenic hPSC line, in which a green fluorescent protein (*GFP*)-encoding sequence had been inserted into an endogenous insulin (*INS*) allele, yielding an INS-GFP-expressing cell line (Micallef et al., 2012). FACS limits the scalability of SCβ-cells production, as it entails a time-consuming procedure on a machine that requires extensive calibration and technical expertise; cells are exposed to additional stress and contamination sources during standby.

Alternatively, magnetic sorting of cell-surface markers shows promise to deplete undesirable cell types and facilitate differentiation towards mature SCβ-cells. The method has been used to remove hPSCs (Fong et al., 2009), isolate anterior definitive endoderm (Mahaddalkar et al., 2020) and enrich for pancreatic progenitors in earlier stages of differentiation (Ameri et al., 2017; Cogger et al., 2017; Kelly et al., 2011). Sorting for surface markers in early stages of pancreatic differentiation generates heterogenous clusters with relatively higher proportions of INS+ SCβ-cells. On the other hand, sorting for late-stage endocrine cell marker CD49a generates clusters enriched for endocrine cells, but its variable inclusion of α-, δ- and pancreatic polypeptide cells (PP-cells) may impede downstream applications such as transplantation (Veres et al., 2019). Recently, Parent et al. (2022) identified monoclonal antibodies against immature SCβ-cells, generating endocrine clusters with improved glucose response and reduced proliferation upon transplantation. Taken together, magnetic sorting of cell-surface markers could offer similar benefits as INS-GFP-based FACS but with greater cost efficiency and scalability. To capitalize on the best of both worlds, we aimed to generate a human embryonic stem cell (hESC) line that expresses a cell-surface marker for which there are available clinical grade isolation reagents to facilitate a specific and scalable enrichment of insulin-expressing SCβ-cells (Arcangeli et al., 2020). Cluster of differentiation 19 (CD19) is a 95 kD transmembrane glycoprotein in the immunoglobulin superfamily. It is expressed across all phases of B-cell development, thus, commonly used as a biomarker for lymphoma diagnosis and leukemia immunotherapies (Wang et al., 2012). CD19 has been established as a reliable cell marker for clinical-grade cell-sorting studies and applications (Groves et al., 2018). Moreover, it has been demonstrated that CD19 is not expressed in pancreatic cells or SCβ-cells (Uhlén et al., 2015). Furthermore, CD19 is the most frequently targeted antigen in the clinical production of chimeric antigen receptor (CAR) T cells (Arcangeli et al., 2020; Zhang et al., 2018; Zhu et al., 2018). In humans, CD19 is an adaptor protein that recruits cytoplasmic signaling proteins to the cell surface, working with CD21 as a complex to establish the intrinsic threshold for B-cell receptor-signaling pathways (Tedder et al., 1997).

In this study, we explored CD19 as a potential cell-surface marker for the enrichment of SCβ-cells. To render a signaling-deficient cell-surface marker, we truncated the cytoplasmic tail at the C-terminus of CD19 and replaced the intracellular domain with the bright red fluorescent protein mScarlet (Bindels et al., 2017; Wang et al., 2012). Using CRISPR/Cas9 technology, we knocked-in added-on the truncated CD19 downstream of the final insulin-coding exon in hESCs. We show that magnetic sorting and reaggregation of immature endocrine cells depleted non-endocrine cells and produced enriched reaggregated SCβ-cells clusters that contain ≤70% CD19-expressing cells. Gene expression analyses confirmed that magnetic sorting enriched for INS-expressing cells over other endocrine cell types. Sorted SCβ-cells also showed a trend of increased connecting (C)-peptide secretion in response to glucose stimulation indicating their enrichment for SCβ-cells. By combining CRISPR/Cas9 technology and a scalable magnetic sorting approach, we can facilitate the large-scale production of SCβ-cells for disease modeling and cell-replacement therapy research.

## RESULTS

### Generation of hESCs with INS-PC1/3-2A-CD19Δ-mScarlet knock-in add-on

**Fig. 1.**
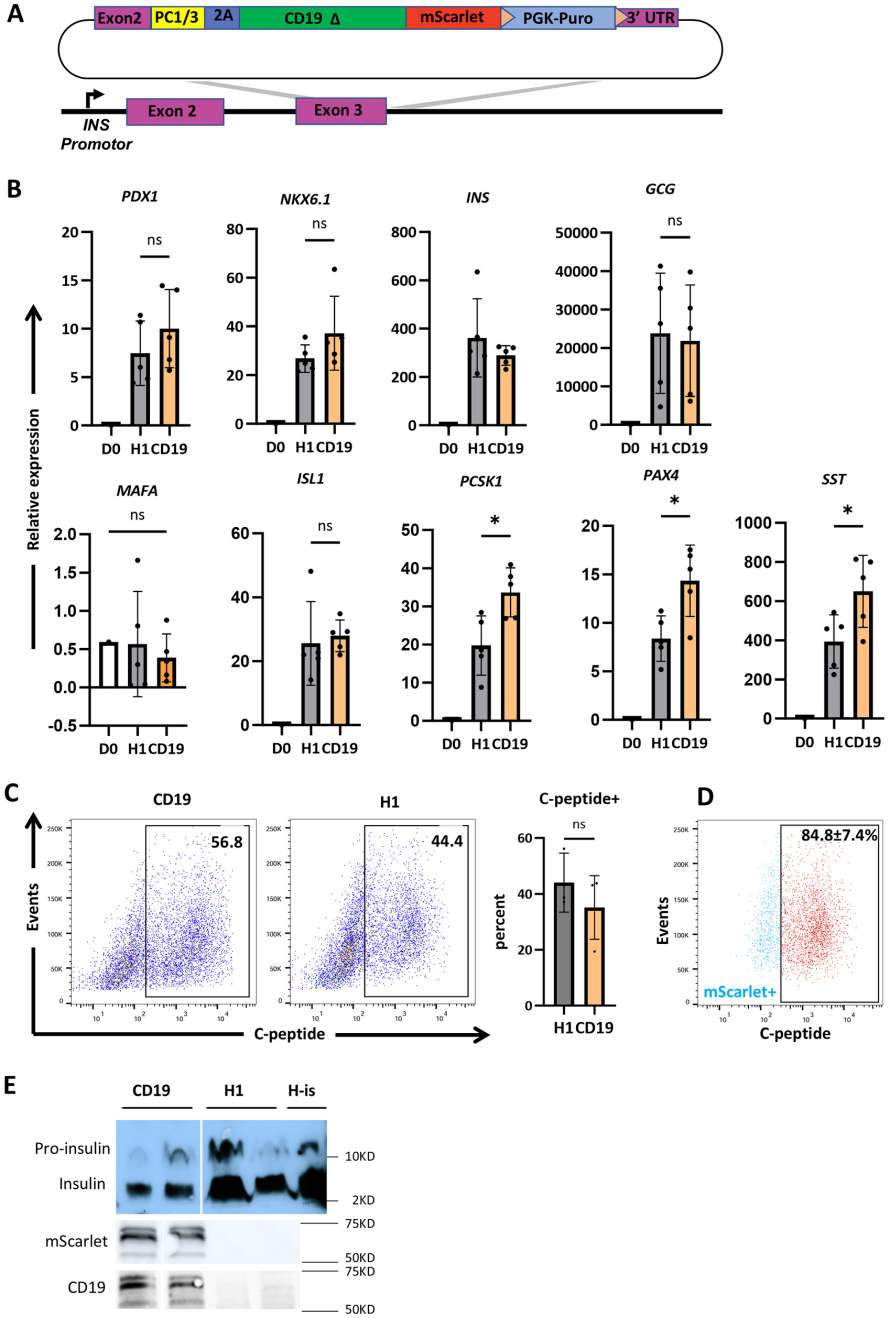
**Generation and characterization of hESCs with INS-2A-CD19-mScarlet.** (A) Schematic overview of the targeting strategy using CRISPR/Cas9 to knock-in add-on 2A-CD19-mScarlet downstream of INS exon 3. The DNA donor vector features a proconvertase (PC) 1/3 recognition site, a 2A fragment to facilitate cleavage, and a fused sequence truncated, signaling-deficient form of CD19 (CD19Δ) and mScarlet red fluorescent protein. (B) Bar graphs showing the results of relative qPCR analysis of cell-specific pancreatic endocrine marker genes in cells of INS-2A-CD19Δ-mScarlet (orange) and H1 (gray) cell lines on day 29 (d29) of differentiation. D0, undifferentiated control. Data represent *n*≥4 independent biological replicates. *P<0.05, unpaired *t*-test. (C) Flow cytometry analysis of C-peptide expression in cells of INS-2A-CD19-mScarlet and H1 cell lines on d29 of differentiation. Bar plots show quantitative representation of the data for INS-2A-CD19Δ-mScarlet (orange) and H1 (gray) cell lines. Data represent *n*=3 independent biological replicates. Statistical significance was assessed using unpaired *t*-test. (D) Representative flow cytometry analysis of mScarlet and C-peptide expression on INS-2A-CD19Δ-mScarlet SCβ-cells. The proportion of C-peptide+ cells (red) within the total mScarlet+ population (blue) is shown in INS-2A-CD19Δ-mScarlet cells. *n*=3 independent biological replicates. (E) Western blot analysis of pro-insulin, insulin, mScarlet and CD19 protein levels on d29-d32 of differentiation for the INS-2A-CD19-mScarlet and H1 cell lines; human islets (H-is) included as a control. D0, INS-2A-CD19Δ-mScarlet hESC line at d0 of differentiation (before starting induction); CD19, INS-2A-CD19Δ-mScarlet line. ns, not significant. Error bars indicate the mean±s.e.m.

To develop a β-cell-specific enrichment strategy that is accessible and efficient, we generated an H1 hESC line expressing the β-cell-surface marker CD19Δ-mScarlet, in which CD19Δ was fused to the bright red fluorescent protein mScarlet, to enable downstream analyses (Gadella et al., 2023). We created a knock-in add-on of the CD19Δ-mScarlet downstream of the insulin-coding region in H1 hESCs (Fig. 1A, Fig. S1A). To prevent CD19Δ-mScarlet from interfering with endogenous SCβ-cell function, CD19 was truncated at the C-terminus to render it signaling deficient (Δ) (Wang et al., 2012). In addition, the knock-in design featured a 2A site to induce ribosomal skipping during translation of INS and CD19Δ-mScarlet, as well as a proprotein convertase (PC) 1/3 site to ensure that residual 2A amino acids are cleaved from the endogenous insulin C-terminus within the secretory granules (Liu et al., 2017). Together, the PC 1/3 site and the 2A fragment separate endogenous INS protein expression from cell-surface CD19Δ-mScarlet expression. To enable clonal selection, the design also featured a Lox-P flanked PGK-puro cassette that was later removed by transfection of a Cre-expressing plasmid.

Puromycin-resistant clones were genotyped using primer pairs that span the 5′ and 3′ homology arms. Out of the 48 clones, 47 (∼98%) were correctly targeted at both the 5′ and 3′ ends, as determined using PCR genotyping; 13 clones were expanded and re-genotyped, and all of them contained the transgene after expansion (Fig. S1B). To characterize whether the insertion targeted one or both alleles, we again used PCR genotyping to distinguish the wild type allele from the modified allele. All 48 clones were heterozygous for the knock-in and all 13 clones identified above remained heterozygous for the CD19Δ-mScarlet knock-in (Fig. S1C). Furthermore, sequencing analysis of the PCR products confirmed precise insertion of INS-2A-CD19Δ-mScarlet and that no mutations were introduced near the Cas9 cleavage site. To assess genomic integrity, we performed quantitative PCR (qPCR)-based profiling on genomic hotspot regions that are commonly altered during reprogramming, including eight common karyotypic abnormalities reported in human ESCs: chromosomes 1q, 4p, 8q, 10p, 12p, 17q, 18q, 20q, Xp. Clones B3, C4 and E3 were karyotypically comparable to the hPSC genomic control, and clone C4 was selected for further differentiation and characterization (Fig. S2).

To evaluate the efficiency of clone C4 hESCs with the INS-PC1/3-2A-CD19Δ-mScarlet knock-in add-on (INS-CD19Δ-mScarlet), we cultured them in 2-dimensional (2D) and then 2-dimensional (3D) (day 0) culture systems (Fig. S3A), and then differentiated these cells for 25–29 days through six stages to derive immature SCβ-cells. qPCR analysis of key islet-specific marker genes, including *PDX1*, *NKX6.1*, *INS*, *GCG*, *MAFA*, *ISL1*, *PCSK1*, *PAX4* and *SST*, was performed on the established knock-in add-on hESC line at day 0 of differentiation, as well as on the parental H1 hESC line (Fig. 1B). qPCR results revealed significantly upregulated levels of islet-specific marker genes in differentiated INS-CD19Δ-mScarlet cells, with comparable gene expression levels for major β-cell marker genes (*PDX1*, *NKX6.1*, *INS*) in INS-CD19Δ-mScarlet and H1-derived SCβ-cells. A slight but significant difference (*P*<0.05) was observed in the expression of *PCSK1*, *PAX4* and *SST* marker genes (Fig. 1B). SCβ-cell differentiation efficiency was assessed by carrying out flow cytometry to assess expression of C-peptide. No significant difference was observed between the two cell lines (Fig. 1C). We used flow cytometry to quantify the number of cells that coexpress mScarlet and C-peptide, which revealed that 84.8±7.4% of Scarlet+ cells also expressed C-peptide (Fig. 1D). To assess the processing of insulin in INS-CD19Δ-mScarlet cells, western blot analysis was conducted for proinsulin and insulin, confirming the presence of both proteins in INS-CD19Δ-mScarlet-derived SCβ-cells at levels comparable to those in H1-derived SCβ-cells (Fig. 1E). In addition, western blot analysis for CD19 and mScarlet revealed the same band pattern, indicating the integrity of these two components in the differentiated INS-CD19Δ-mScarlet hESC line. Our CD19-mScarlet construct was designed to produce a 64 kDa protein – which aligns with the observed band. The upper band is likely due to different glycosylation states of truncated CD19 (Fig. 1E), as previously reported in other studies (Bagashev et al., 2018; Heard et al., 2022).

In summary, we identified a targeting approach that allows highly efficient introduction of transgenes downstream the insulin coding region in human embryonic stem cells. Using this strategy, we generated an INS-CD19Δ-mScarlet hESC line that successfully passed a multi-step quality-control workflow, including genetic evaluation, SCβ-cell differentiation assays and insulin production assessment.

### CD19-mScarlet localizes to the cell surface of SCβ-cells

**Fig. 2.**
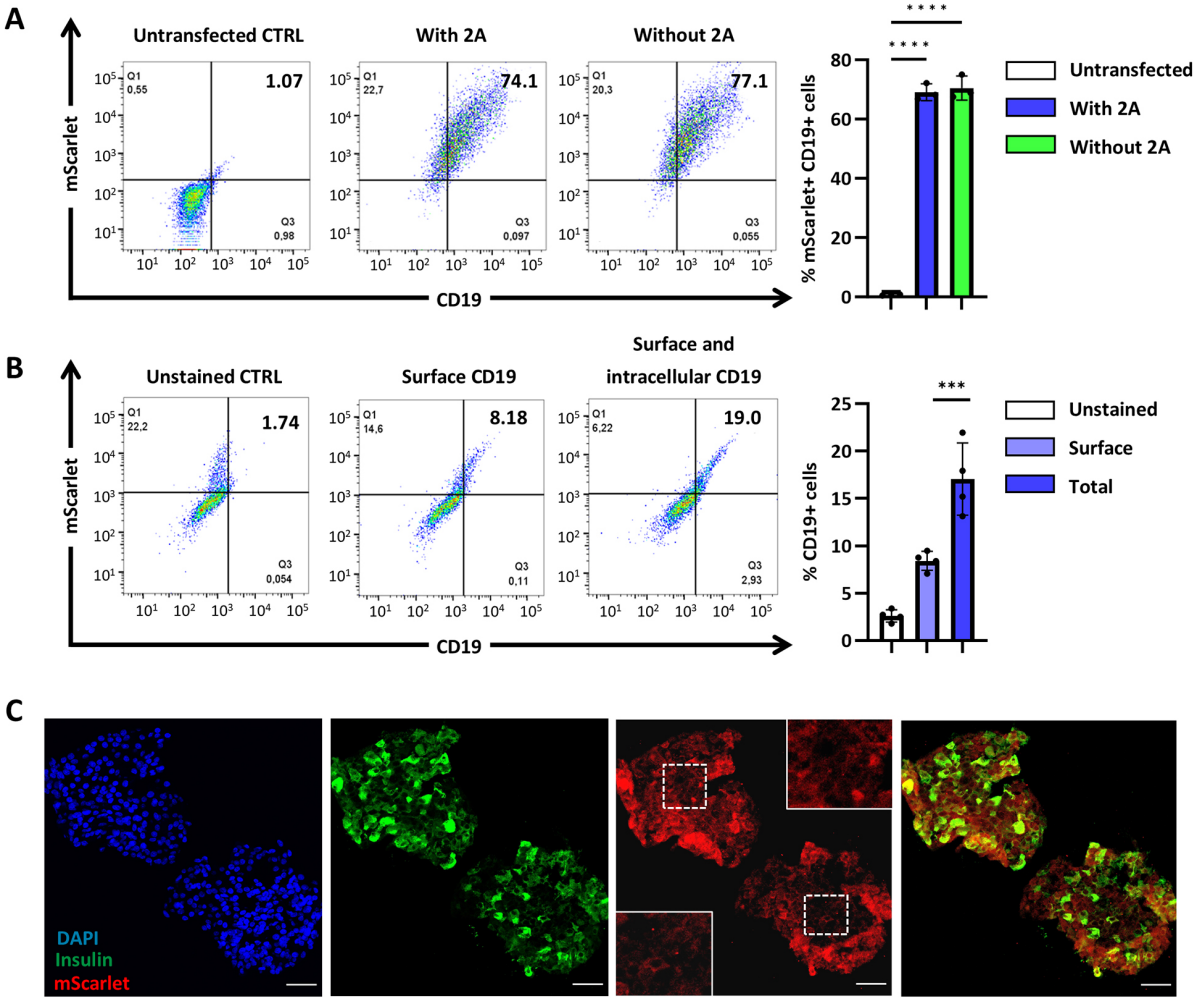
**CD19Δ-mScarlet localizes to the cell surface of SCβ-cells.** (A) HEK293 cells express CD19Δ-mScarlet 72 h post transfection. HEK293 cells were transfected with the CD19Δ-mScarlet construct with or without 2A at the N-terminus. Cells were dissociated and assayed for CD19 and mScarlet expression with flow cytometry after 72 h. *n*=3 independent biological replicates. (B) Representative flow cytometry analysis of CD19 expression on unstained, surface stained, and surface and intracellularly stained d25 SCβ-cells. *n*=4 independent biological replicates. ***P<0.001, ****P<0.0001 (one-way ANOVA followed by Tukey's multiple comparisons test). Error bars indicate the mean±s.e.m. (C) Immunofluorescence images of unsorted stage 6, d25 immature INS-2A-CD19Δ-mScarlet SCβ-cell clusters expressing INS-2A-CD19Δ-mScarlet (red). Third image: Boxed areas on the left and right indicate magnified representative views of bottom left and top right, respectively. Staining was against insulin (green); nuclei of clustered cells were stained with DAPI (blue). Scale bars: 50 µm.

In addition to generating the above-described cell lines we assessed the efficiency of CD19Δ-mScarlet protein expression and localization to the cell surface. For that, we subcloned the DNA sequences encoding CD19Δ-mScarlet or 2A-CD19Δ-mScarlet into plasmid pCDNA3.1+ and used transient transfection to express them in HEK293 cells. On average, 70.5% of transfected HEK293 cells expressed both mScarlet and cell-surface CD19Δ, as assessed by flow cytometry (Fig. 2A). We found no differences in CD19Δ-mScarlet expression or localization between the two constructs. These experiments demonstrated that the signal peptide in CD19Δ-mScarlet is functional and not affected by the presence of residual 2A sequence at its N-terminus.

To determine whether CD19Δ-mScarlet localizes to the cell surface of SCβ-cells, we compared surface and intracellular expression of CD19 protein in cells differentiated for 25 days (hereafter referred to as d25 SCβ-cells). Briefly, dissociated SCβ-cells were stained for surface CD19, fixed and permeabilized, then stained again for surface and intracellular CD19. Flow cytometry analysis showed that, in bulk, half of all detectable CD19Δ-mScarlet protein was located on the cell surface (Fig. 2B). Live confocal imaging confirmed that CD19Δ-mScarlet colocalizes with the cell membrane in regions of the SCβ-cell cluster that abundantly express CD19Δ-mScarlet (Fig. S4A,B, arrows). Furthermore, immunofluorescence analysis showed insulin expression in CD19Δ-mScarlet-expressing cells of the same cluster (Fig. 2C).

### Enrichment of CD49a on SCβ-cells by using magnetic-activated cell sorting

**Fig. 3.**
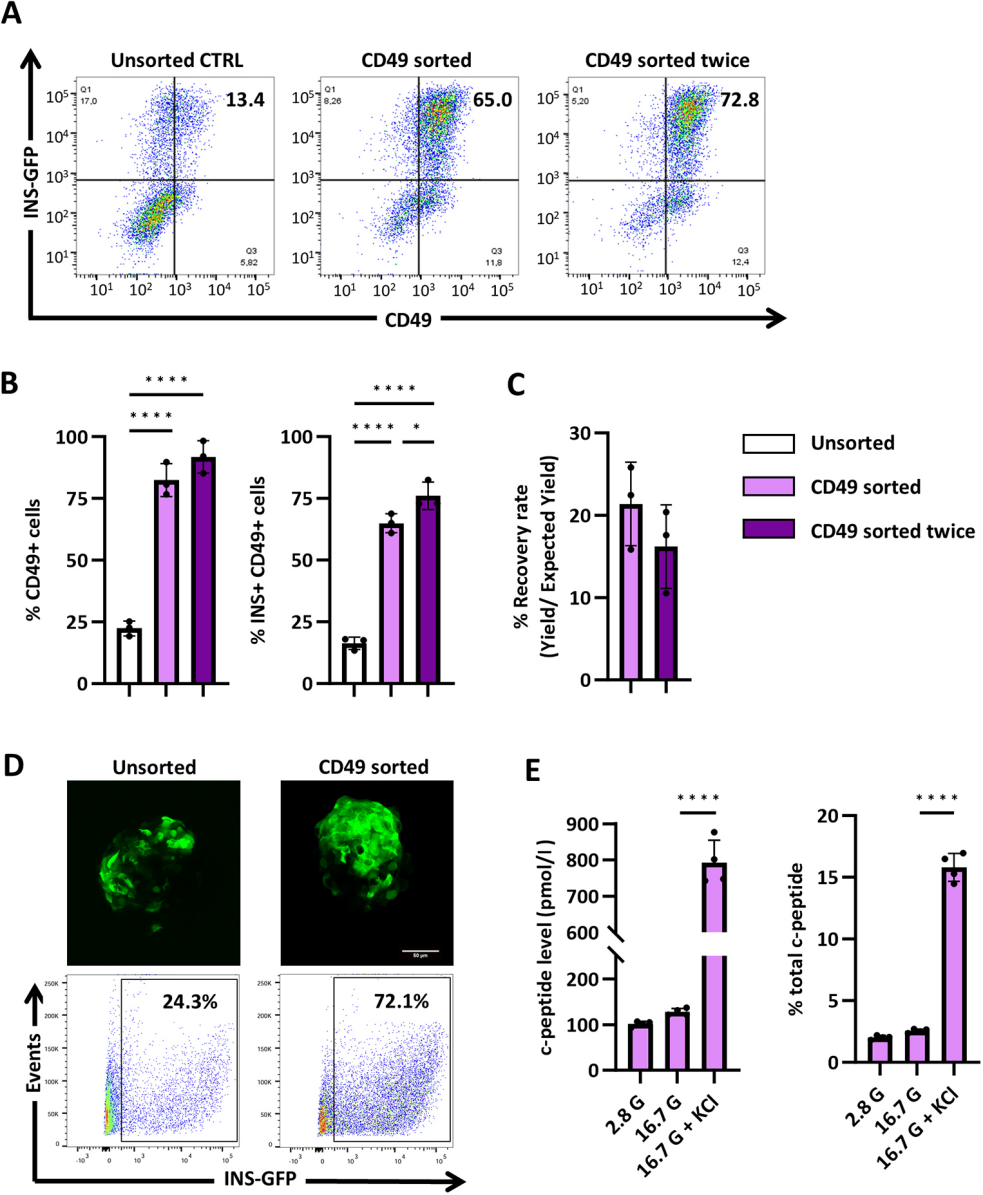
**MACS enriches CD49+ cells in immature INS-GFP SCβ-cells.** (A) Representative flow cytometry analysis of CD49 expression on day 25 in SCβ-cells that were unsorted (Unsorted CTRL), sorted once (CD49 once), or sorted twice (CD49 sorted twice). (B) Bar plot quantification of flow cytometry data showing the proportions of CD49^+^ cells and CD49^+^ INS-GFP^+^ cells in the Unsorted, CD45 sorted, and CD45 sorted twice groups. *n*=3 independent biological replicates. *P<0.05, ****P<0.0001 (one-way ANOVA followed by Tukey's multiple comparisons test). (C) Recovery rate was calculated by dividing yield by expected yield. Statistical significance was determined with the unpaired *t*-test. *n*=3 independent biological replicates. (D) Live confocal imaging of unsorted and sorted stage 6, d35 immature INS-2A-CD19Δ-mScarlet SCβ-cell clusters expressing INS-GFP (green). Scale bar: 50 µm. Quantitative presentation of the flow cytometry data from panel A, highlighting the percentage of GFP-expressing cells in unsorted and CD49-sorted populations. (E) Static secretion of C-peptide in response to stimulation with 2.8 mM glucose, 16.7 mM glucose and 40 mM KCl in an *in vitro* assay for sorted stage 6, d35 immature INS-2A-CD19Δ-mScarlet SCβ-cell clusters. Total C-peptide was collected by ethanol acid extraction. *n*=4 independent biological replicates. ****P<0.0001 (one-way ANOVA followed by Tukey's multiple comparisons test). Error bars indicate the mean±s.e.m.

We evaluated the efficiency of magnetic-activated cell sorting (MACS) by reproducing the enrichment of CD49a-expressing stem cell-derived islets cells described by Veres et al. (2019). CD49a sorting entailed an indirect binding approach, i.e. CD49a epitopes were bound to CD49a-PE antibodies followed by binding to anti-PE microbeads for magnetic selection. We used an INS-GFP hESC line that had been previously characterized and collected cells for flow cytometry analysis at each stage of MACS (Novakovsky et al., 2023). We further characterized our INS-GFP hESC line by using flow cytometry analysis of INS-GFP and C-peptide expression during SCβ-cell differentiation. Comparison with the parental H1 line revealed that the average differentiation efficiency, based on C-peptide+ cells, was 38.5% for the H1 line and 35.94% for the INS-GFP line, with no significant difference between the two. Moreover, GFP+ cells closely represented the C-peptide+ cell population (Fig. S5A). Consistent with the results reported by Veres et al. (2019), MACS of SCβ-cell clusters enriched the INS-GFP+ sCD49a+ population by ≤75%, representing a more than threefold increase compared with unsorted clusters (Fig. 3A,B). An optional enrichment step with a second magnetic column further increased the INS+ sCD49a+ proportion by ∼10% (Fig. 3A,B). The recovery rate (see ‘Magnetic-activated cell sorting for CD49a and CD19’ section in the Materials and Methods for the calculation method), showed a decreasing trend as the number of column enrichment steps increased (Fig. 3C). The CD49a-sorted cells were seeded in pre-patterned AggreWell plates (StemCell Technologies Inc.) for 3 days and reaggregated into clusters of consistent size (∼100−200 µm). Cell clusters were transferred to suspension culture for an additional 7 days. After extended culture, the reaggregated SCβ-cells retained high GFP expression (Fig. 3D). This suggests that CD49a magnetic sorting enriched for a viable population of INS-GFP-expressing cells, while the presence of microbead attachments did not affect downstream reaggregation of cell clusters.

Reaggregated CD49a-sorted clusters were cultured for 10 days and collected on d35 for functional analysis. To assess C-peptide secretion in response to glucose stimulation, these CD49a-enriched clusters were incubated in 2.8 mM glucose, 16.7 mM glucose and 16.7 mM glucose with 30 mM KCl for 1 h consecutively. Supernatants at each stage were collected for C-peptide quantification via ELISA. CD49a-sorted clusters showed glucose-stimulated C-peptide secretion (Fig. 3E). Taken together, these results confirm that MACS can reproducibly enrich for surface markers expressed in stem cell-derived islet cells.

### CD19-enriched SCβ-cells show improved INS expression and C-peptide secretory profile

**Fig. 4.**
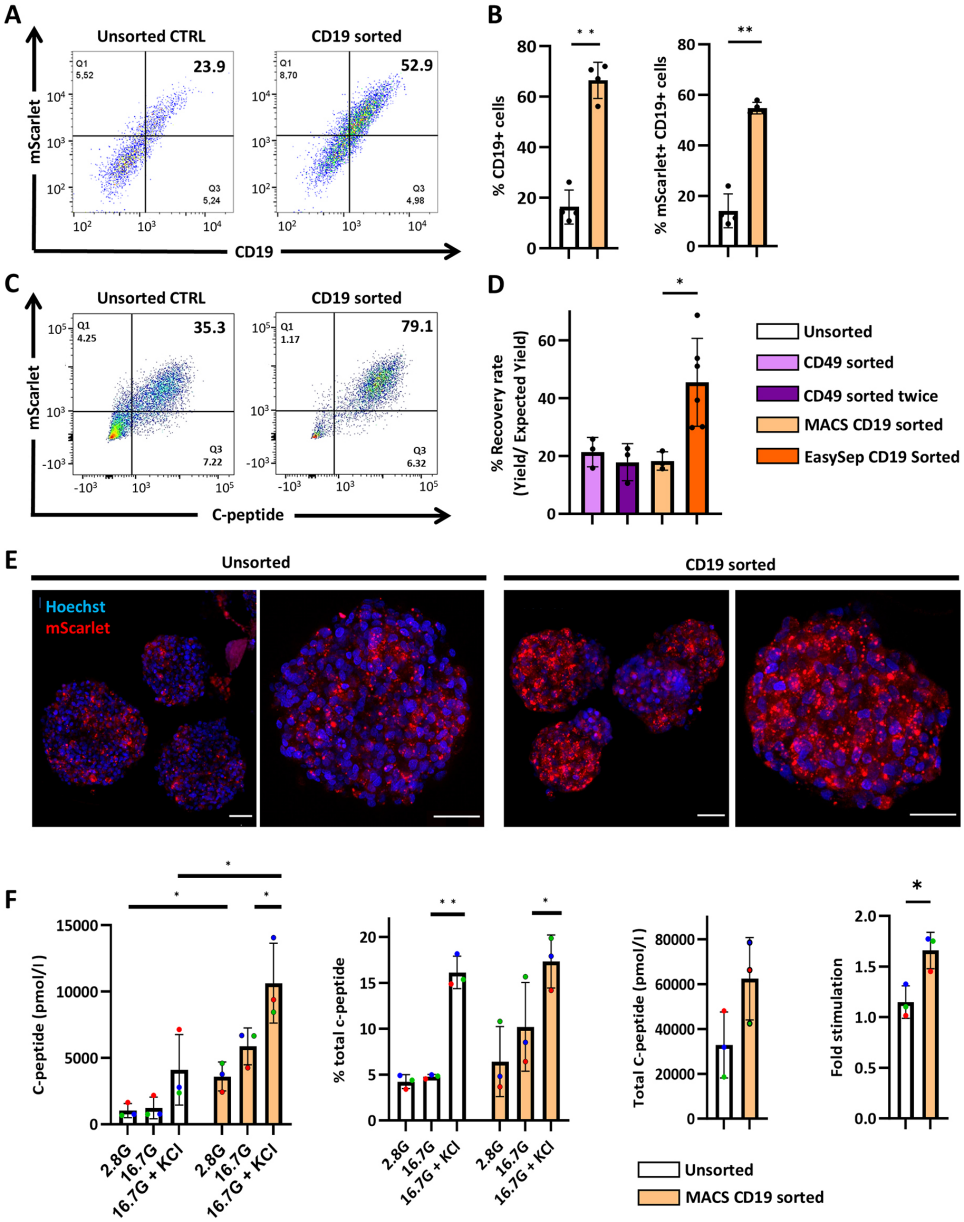
**MACS enriches CD19+ mScarlet+ and C-peptide+ cells from immature INS-2A-CD19Δ-mScarlet SCβ-cells.** (A) Representative flow cytometry analysis of CD19 expression on d35 INS-2A-CD19Δ-mScarlet SCβ-cells that were unsorted and CD19 sorted. (B) Bar plot quantification of flow cytometry data showing the proportions of CD19+ cells and CD19+ mScarlet+ cells in the unsorted and MACS CD19 sorted groups. *n*=3; black circles indicate individual biological replicates. **P<0.01 (one-way ANOVA followed by Tukey's multiple comparisons test.). (C) Representative flow cytometry analysis of C-peptide expression on d25 SCβ-cells that were unsorted and CD19 sorted. *n*=2 independent biological replicates. (D) Recovery rate was calculated by dividing yield by expected yield. Statistical significance was determined with the unpaired *t*-test. *n*=3; black circles indicate individual biological replicates. *P<0.05 (one-way ANOVA with multiple comparisons). (E) Live confocal imaging of unsorted and CD19 sorted stage 6, d35 immature INS-2A-CD19Δ-mScarlet SCβ-cell clusters expressing INS-2A-CD19-ΔmScarlet (red). Scale bars: 50 µm. (F) Static secretion of C-peptide in response to stimulation with 2.8 mM glucose, 16.7 mM glucose and 40 mM KCl in an *in vitro* assay. Total C-peptide was collected by ethanol acid extraction. Fold-stimulation was calculated as C-peptide secretion at high glucose divided by C-peptide secretion at low glucose. *n*=3; each colored circle denotes a biological replicate, with identical colors indicating the same sample. *P<0.05, **P<0.01 (one-way ANOVA followed by Tukey's multiple comparisons test). Error bars indicate the mean±s.e.m.

Compared to CD49a MACS, CD19Δ MACS used a direct binding approach, in which d25 INS-2A-CD19Δ-mScarlet SCβ-cells were enzymatically dissociated and directly labelled with CD19-specific microbeads. Similar to the pattern previously observed with CD49a sorting, CD19-sorted cell clusters showed ∼50% more CD19Δ-expressing cells than unsorted SCβ-cell clusters (Fig. 4A), again this represented a more than threefold enrichment. This trend was consistent in both CD19Δ and mScarlet-expressing cell populations as expected (Fig. 4B). Also, sorting led to the obvious enrichment of the mScarlet+C-peptide+ cell population (Fig. 4C). The recovery rate of CD19Δ-sorting was comparable to CD49a sorting, both before and after a second optional column enrichment (Fig. 4D). We compared commercially available magnetic sorting kits and found that the EasySep CD19 Positive Selection Kit (StemCell Technologies Inc.) yielded a recovery rate of ≤70%.

The CD19Δ-sorted cells were seeded in AggreWell plates for 3 days to generate clusters of consistent size (∼100-200 μm). Cell clusters were transferred to suspension culture for an additional 7 days before collection for live confocal imaging. While we observed the the mScarlet+ population at different days of differentiation (between days 25 and 35) to range from about 23% to about 46% in the unsorted population (Figs 2B, 4A,C and Fig. S5B), sorting enriched this population to about 61–80% (Fig. 4A,C). This enrichment is also apparent visually in CD19Δ-sorted cell clusters (Fig. 4E). As assessed by regular fluorescence and confocal microscopy, mScarlet expression remained stable throughout differentiation (Fig. S3B,C).

To determine the functionality of our enriched and reaggregated SCβ-cell clusters, we performed glucose-stimulated C-peptide secretion to test 50 CD19Δ-sorted SCβ-cell clusters in their response to glucose and KCl stimulation. Compared to unsorted clusters, both basal and total C-peptide secretion significantly increased in CD19Δ-sorted clusters by ∼3.5-fold and ∼2.5-fold, respectively. (*P*<0.05) (Fig. 4F). This increase indicates either a higher proportion of INS-expressing cells within the clusters, confirming enrichment of β-cells after sorting, or an overall enhancement in functionality of the sorted cell population. Interestingly, reaggregated CD19Δ-sorted SCβ-cells showed a trend of increased C-peptide secretion in response to 16.7 mM glucose stimulation and had significantly improved stimulation index (Fig. 4F).

**Fig. 5.**
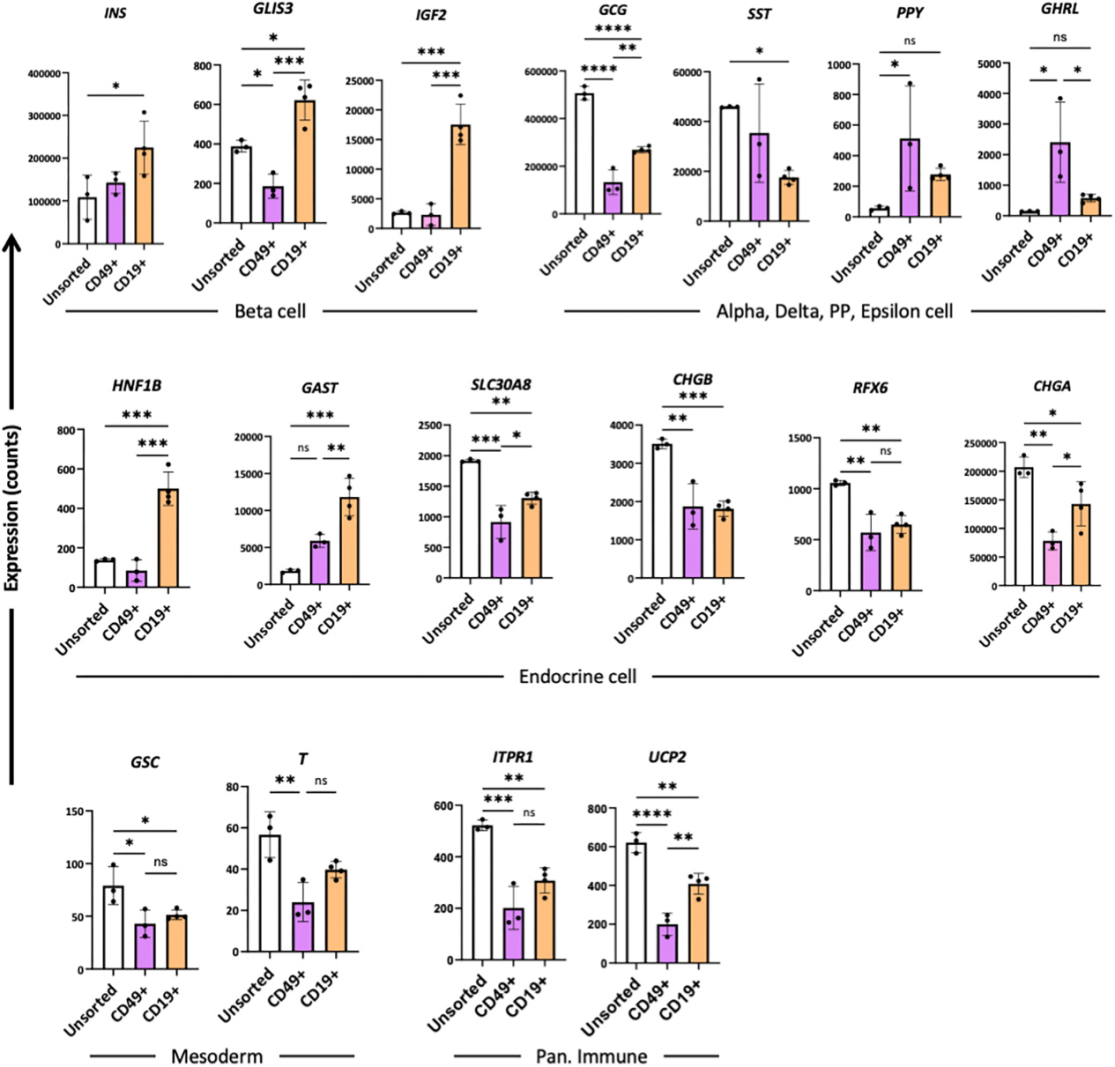
**Gene expression analysis of unsorted, CD49 sorted and CD19 sorted SCβ-cells using NanoString analysis.** Plotted are the expression counts of selected genes as indicated, with key marker genes from islets (top), endocrine (middle), mesodermal lineage and pancreatic immune cells (bottom). CD49+, CD49 sorted SCβ-cells; CD19+, CD19 sorted SCβ-cells. *n*=3 independent biological replicates. *P<0.05, **P<0.01, ***P<0.001, ****P<0.0001 (one-way ANOVA followed by Tukey's multiple comparisons test.); ns, not significant. Error bars indicate the mean±s.e.m.

Cells of INS-GFP and INS-2A-CD19Δ-mScarlet hESC lines were differentiated up to d25, sorted for CD49a and CD19, respectively, followed by NanoString gene expression analysis of 149 annotated genes categorized into 16 groups (Fig. S6A). This revealed that sorting with either CD49 or CD19 led to significant changes in the expression of 48 genes compared to the unsorted group. Notably, 19 genes were differentially expressed between CD49-sorted and CD19-enriched cells, of which >25% were annotated as endocrine markers and <6% as exocrine or ductal cell markers (Fig. S6B). Gene expression analyses confirmed increased *INS* expression and decreased *GCG* (alpha cell marker) and *SST* (delta cell marker) expression in CD19Δ-sorted clusters, suggesting that our magnetic sorting strategy enriched for the cell population that express higher levels of *INS* compared with those of alpha and delta marker genes (Fig. 5). When comparing CD49a- and CD19Δ-cell sorting, the latter resulted in a population expressing higher levels of *GLIS3* and *IGF2* – both of which are β-cell-specific marker genes, and lower levels of *GHRL* – the marker gene of epsilon cells (Fig. 5). Interestingly, CD19Δ-sorted cell clusters expressed significantly higher levels of IGF2 than unsorted or CD49a-sorted clusters (Fig. 5). This may be related to the enrichment of INS-expressing cells, as IGF2 and INS share a promoter and are often expressed as INS-IGF2 hybrid transcripts in the human pancreas (Monk et al., 2006). IGF2 is a potent growth factor recognized for its anti-inflammatory and anti-apoptotic effects within the pancreatic islets, potentially facilitating protective conditions for islet transplantation (Calderari et al., 2007; Hughes et al., 2013; Sandovici et al., 2021). While the expression of certain endocrine markers, such as HNF1B and GAST, was significantly upregulated in the CD19-sorted cell population, other marker protein, including SLC30A8, CHGB, RFX6 and CHGA, showed a significant decrease in the sorted cell population (Fig. 5). Decreased expression of CHGA and CHGB in sorted clusters is likely due to significant depletion of GCG-expressing cells (Augsornworawat et al., 2020; Veres et al., 2019). Mesodermal marker genes and genes associated with pancreatic immune cells also showed a significant decrease in both sorted groups. Taken together, these data demonstrate that the magnetic sorting method developed by using the INS-2A-CD19Δ-mScarlet hESC line generates enriched SCβ-cell clusters with improved yield, purity and function over other approaches.

## DISCUSSION

SCβ-cell-replacement therapy is a promising alternative to existing treatment options for T1D. However, heterogeneity within SCβ-cell cultures remains problematic for graft safety and long-term function. Here, we developed a SCβ-cell-specific selection protocol that does not rely on fluorescent reporters of insulin expression. Using CRISPR/Cas9, we knocked-in added-on a truncated form of CD19 downstream of *INS* exon 3 in hESCs. We confirmed cell-surface expression of CD19Δ-mScarlet. Importantly, we developed and optimized a magnetic sorting method for INS-CD19Δ-expressing cells, forming enriched SCβ-cell clusters.

To generate a reporter cell line that faithfully reflects INS expression, Micallef et al. (2012) used homologous recombination to replace one of the INS alleles with GFP. In contrast, our INS-2A-CD19Δ-mScarlet cell line featured a heterozygous knock-in add-on. The DNA vector includes a PC1/3 site and a 2A fragment to separate endogenous INS activity from cell-surface expression of CD19Δ-mScarlet. This design allows both copies of the *INS* allele to retain endogenous function, enabling the reaggregated SCβ-cell to mature functionally and respond more physiologically to external stimuli.

Intracellular staining for CD19Δ shows that only 50% of all detectable CD19Δ protein in SCβ-cells is on the cell surface. One possible explanation is that, even with incorporation of PC1/3 and 2A within the donor construct, a proportion of CD19Δ-mScarlet did not fold properly and was targeted to the lysosomal compartment for degradation (Fig. S4A). Although we confirmed stable and efficient cell-surface expression of CD19Δ-mScarlet in HEK293 cells, we cannot exclude the possibility that improperly folded CD19Δ-mScarlet is targeted to lysosomes in immature SCβ-cells. Future iterations of the INS-2A-CD19Δ-mScarlet cell line may incorporate addition of a few glycine-serine linker sequences between CD19Δ and mScarlet to allow more of CD19Δ protein to properly fold and target the cell membrane (Van Rosmalen et al., 2017).

Previous studies have reported magnetic sorting strategies that enrich for anterior definitive endoderm (Mahaddalkar et al., 2020) and pancreatic progenitors (Ameri et al., 2017; Cogger et al., 2017; Kelly et al., 2011). Sorting for surface markers at early stages of pancreatic differentiation has generated SCβ-cell clusters that remain heterogenous with <60% of insulin-expressing cells. Parent et al. (2022) introduced monoclonal antibodies (mAbs) targeting immature SCβ-cells, achieving >70% insulin-expressing cells with C-peptide+ cell recovery rates of 30-50%. However, their approach revealed variability in the efficiency of different mAbs when applied to hESC- versus iPSC-derived SCβ-cells, likely due to differences in timing and levels of epitope expression across cell lines. This variability suggests that additional optimization is required for sorting islet cells from diverse hPSC lines, potentially complicating its broader application. On the other hand, sorting for endocrine cell marker CD49a generated clusters that contain ≤80% SCβ-cells with improved response to glucose *in vitro* (Veres et al., 2019). In our hands, CD49a sorting generated clusters with ∼65% INS-GFP- and CD49a-expressing cells; but these cells showed a limited response of C-peptide to glucose stimulation. The observed difference in the percentage of CD49a+ cell populations may stem from several factors. First, differences between the cell lines used could play a role, as Veres et al. utilized the HUES8 hESC line (Veres et al., 2019), whereas our study employed the INS-GFP cell line derived from the H1 hESC line. Second, variability in the differentiation protocols is another potential source. While both studies followed a six-stage differentiation protocol, there were notable differences in culture systems, parameters, used morphogens and timings, particularly from stage 4 onwards. Veres et al. (2019) collected cells from day 27 onwards, whereas we collected aggregates up to day 25. Finally, technical differences in MACS sorting conditions may contribute. Although the same antibodies and similar MACS sorting systems were used by Veres et al. (2019) and in our study, slight variations in antibody staining protocols, cell densities during sorting, and other procedural details could have influenced the outcomes.

In contrast to endogenous endocrine markers, such as CD49a, we chose to sort for a surface marker that is specific for INS-expressing cells. We observed an improvement in the stimulation index from CD19Δ-sorted clusters. Gene expression analyses further indicate that CD19Δ-sorting was more effective than CD49a for enrichment of *INS*-expressing cells and depletion of *SST*-, *PPY*- and *GHRL*-expressing cell populations; although, future studies might aim to analyze these differentially expressed genes at protein level to confirm these observations. Despite significant enrichment, both CD49a- and CD19Δ-sorted clusters remained heterogenous, containing a proportion of INS-negative cells. This may be related to the inclusion of polyhormonal cells that express *INS* temporarily before resolving into monohormonal *GCG*- or *SST*-expressing cells upon further maturation (Alvarez-Dominguez et al., 2020; Mar et al., 2025). Future attempts of magnetic sorting might consider extending the initial culture time to 35 days, when a large majority of polyhormonal cells have differentiated further into more-defined cell types (Mar et al., 2025).

To further enrich for *INS*-expressing SCβ-cells, sequential magnetic selections may be employed at the expense of cell yield (Fig. 4D). The sequential separations may feature two rounds of positive CD19Δ enrichment. They may also positively select for two different SCβ-cell markers of interest, such as a combination of CD49a labelling followed by CD19Δ labeling. Alternatively, separation could start by negatively selecting for non-SCβ-cell-surface markers, such as GP2 (Ameri et al., 2017), followed by CD19Δ-based positive selection.

In summary, we developed a SCβ-cell-specific selection protocol and optimized enrichment conditions to facilitate efficient recovery of *INS*-expressing cells from hESC pancreatic differentiations. Our study provides a proof-of-concept that MACS of genetically engineered cell-surface markers may enable the large-scale production of functional islet-like clusters suitable for disease modeling and cell-replacement therapies for patients with T1D. However, given that CD19 is a clinically relevant B-cell marker and a common target for CAR-T therapies, its use may pose regulatory considerations for clinical application.

## MATERIALS AND METHODS

### Regulatory approvals

The WA01 hESC line was generously provided by WiCell (Madison, WI, USA). Experiments were approved by the University of British Columbia (UBC)/Children's and Women's Health Centre of British Columbia Research Ethics Board (hESCs: H09-00676) and the CIHR Stem Cell Oversight Committee. Human islets were obtained under informed consent, and isolated and provided by the Alberta Diabetes Institute (ADI) Clinical IsletCore Laboratory (University of Alberta, Edmonton, Canada).

### DNA-targeting constructs

A guide RNA (gRNA) sequence targeting the 3′ end of the insulin-coding sequence (3′-GTGCAACTAGACGCAGCCCGC-5′) was cloned into the pCCC CRISPR/Cas9 vector to create pCCC-766/767 as previously described (Krentz et al., 2014; Novakovsky et al., 2023). For the donor vector we cloned 800-bp-long homology arms into the KpnI/NheI and AscI/NotI restriction sites of the Addgene vector OCT4-2A-eGFP-PGK-Puro (Addgene #31938; Hockemeyer et al., 2011). We then removed the stop codon, gRNA-binding site and PAM sequence from the homology arms to prevent re-cleavage of targeted DNA. We next synthesized a Geneblock (IDT DNA Technologies) containing a human codon-optimized version of PCSK1/3-P2A-CD19Δ-mScarlet and inserted it into the NheI/EcoRI restriction sites to complete the donor vector (Fig. S1). The P2A site was included to allow ribosomal skipping and production of membrane-targeted CD19Δ-mScarlet downstream of insulin, while the PCSK1/3 site was included to insure removal of any residual 2A protein fragments from the C-terminal insulin sequence within the secretory granule (Kim et al., 2011). CD19Δ-mScarlet contained a membrane targeting signal at the N-terminus and a series of stop codons at the C-terminus but lacked an exogenous polyadenylation sequence. Prior to transfection, we fully sequenced the cloned regions using Sanger sequencing.

To test expression of the codon-optimized version of CD19Δ-mScarlet in HEK293 cells, we subcloned fragments containing only CD19Δ-mScarlet or 2A-CD19Δ-mScarlet into the NheI/EcoRI restriction sites of pCDNA3.1+ (Invitrogen) by using PCR.

### Transfection of HEK293 cells

To generate transgenic HEK293, cells were seeded at a density of 0.8×10^6^ cells per well in a six-well suspension plate. 4 µg of the P2A-CD19Δ-mScarlet or CD19Δ-mScarlet construct was transfected using Lipofectamine 2000 (Life Technologies) as per manufacturer's recommendations. Cells were cultured in DMEM (Gibco) supplemented with 10% fetal bovine serum (FBS; Gibco), and penicillin/streptomycin (Pen/Strep; HyClone) in a humidified incubator (5% CO_2_, 37°C) for 72 h before dissociation and staining for flow cytometry analysis.

### Maintenance of human embryonic stem cells

Undifferentiated H1 (WA01; XY) human embryonic stem cells (hESCs) were maintained on Geltrex-coated plates (1:100 in DMEM/F12; Gibco) in StemFlex Medium (Gibco) or mTeSR Plus (StemCell Technologies Inc.) in a humidified incubator (5% CO_2_, 37°C). Cells were passaged every 4 days by non-enzymatic dissociation with stem cell selection and passaging reagent (ReLeSR, StemCell Technologies Inc.) and plated at a density of 5.0×10^5^ per 60-mm plate.

### Generation of INS-2A-CD19Δ-mScarlet hESC line

To generate the INS-2A-CD19Δ-mScarlet knock-in add-on hESC line, CRISPR/Cas9 was used as previously described (Krentz et al., 2014). At 4-6 h prior to electroporation, H1 hESC culture medium was replaced with fresh StemFlex medium (Gibco). hESCs were washed with PBS lacking Ca^2+/^Mg^2+^ (PBS−) before dissociation with Accutase cell-detachment solution (StemCell Technologies Inc.) for 5 min at 37°C. Following detachment, cells were resuspended in PBS supplemented with Ca^2+^/Mg^2+^ (PBS++) and centrifuged at 200 g for 5 min. Then, 5×10^6^ cells were resuspended in 0.7 ml PBS++, transferred into a 0.4-cm electroporation cuvette with 40 µg INS-2A-CD19Δ-mScarlet donor plasmid and 15 µg pCCC-LL766/767, and electroporated using a Bio-Rad Laboratories Gene Pulser II system (250 V, 500 µF, infinite resistance, time constant <10 ms). After electroporation, cells were resuspended in Stemflex supplemented with 1 mM of the ROCK inhibitor Y-27632 (StemCell Technologies Inc.) and plated onto a 60-mm Geltrex-coated tissue culture dishes. Cells were allowed to recover for up to four days with daily changes of Stemflex medium before selecting with 0.25 µg/ml puromycin (Sigma Aldrich). Colonies were transferred into 96-well Lamin-521-coated plates (13.3 µg/ml in PBS++; Biolamina) within 10 days of electroporation, by manually scraping and pipetting colonies off the plate and into a well containing 100 µl Stemflex with 10 µM Y-27632. Once clones were close to confluency, cells were replica-plated onto three plates: one to genotype analysis, one to freeze and one to expand correctly targeted clones. Genomic DNA was extracted using QuickExtract (Lucigen) and the following primers, spanning the 5′ and 3′ homology arms were used to genotype: 5′F LL1059: 3′-CTTTGAGGCACTGCAAAACTGCGT-5′; 5′R LL688: 3′-TGACCGCAGATTCAAGTGTT-5′; 3′F LL789: 3′-GAAGGATTGGAGCTACGGGG-5′; 3′R LL790: 3′-AGCTCATGGTGCCATCTGAC-5′.

To enable clonal selection, the INS-2A-CD19Δ-mScarlet donor plasmid also featured a Lox-P-flanked PGK-puro cassette that was later removed by transfection of Cre-expressing plasmids. Briefly, 4-6 h prior to electroporation, culture medium of INS-2A-CD19Δ-mScarlet hESC clones B3, C4 and E3 was replaced with fresh StemFlex medium. hESCs were washed with PBS− before dissociation with Accutase solution for 5 min at 37°C. Detached cells were resuspended in PBS++ and centrifuged at 200 ***g*** for 5 min. 5×10^6^ cells were resuspended in 0.7 ml PBS++, transferred to a 0.4-cm electroporation cuvette with 15 µg of pCAGGS−GFPCre (Krentz et al., 2014) plasmid DNA, and electroporated using a Bio-Rad Laboratories Gene Pulser II system (250 V, 500 µF, infinite resistance, time constant <10 ms). Electroporated cells were resuspended in Stemflex with 1 mM Y-27632 and plated onto 60-mm Geltrex-coated tissue culture dishes for recovery.

After 36-48 h, cells were sorted for GFP-Cre expression using the BD FACSAria™ II Cell Sorter. 4-6 h prior to FACS, culture media of hESC clones B3, C4 and E3 were replaced with fresh StemFlex. hESCs were washed with PBS− before dissociation with Accutase solution for 5 min at 37°C. Detached cells were resuspended in 600 μl of PBS++ with ROCK inhibitor and filtered through a 40 µm FACS tube. GFP+ cells were sorted into Stemflex with 1 mM Y-27632 and 1× PenStrep, then plated onto a 60 mm Geltrex-coated tissue culture dish. PGK-Puro cassette removal was verified by culturing sorted cells with 0.25 µg/ml Puromycin and by genotyping.

### Karyotyping and genomic abnormalities analysis

Genomic integrity was assessed with the hPSC Genetic Analysis Kit (StemCell Technologies, #07550) according to the manufacturer's instructions. Briefly, 5 ng of genomic DNA was mixed with a ROX reference dye (Thermo Fisher Scientific) and double-quenched probes tagged with 5-FAM. The probes represented eight common karyotypic abnormalities that have been reported to arise in cloned pluripotent cells at chromosome locations chr 1q, chr 8q, chr 10p, chr 12p, chr 17q, chr 18q, chr 20q or chr Xp. Sample-probe mixes were analyzed on a ViiA^TM^7 PCR System (ThermoFisher Scientific). The results were normalized to the copy number of a control region within chr 4p and analyzed using the ΔΔCt method.

### *In vitro* differentiation of hESCs

To initiate differentiation, we washed 80-90% confluent cultures with PBS− and dissociated into a single-cell suspension using Accutase solution (8 min, 37°C). Single cells were centrifuged at 200 ***g*** for 5 min and resuspended in StemFlex supplemented with 10 µM Y-27632. We seeded 5.5×10^6^ cells per 5.5 ml of mTeSR+ into each well of six-well suspension plates. The plates were incubated overnight at 37 °C and 5% CO_2_ on an orbital shaker (25 mm orbit; Celltron, Infors HT) set at 95 rpm. The next morning, spheroids were washed once with PBS++ (Gibco) and resuspended in day (d) 0 differentiation medium (see Table S1), with a final volume of 5.5-ml per well. Spheroids were fed daily by removing spent medium and replenishing with 5.5 ml of fresh medium. Details of all differentiation media are summarized in Table S1.

### Magnetic-activated cell sorting for CD49a and CD19

d25 immature SCβ-cell clusters were washed twice with PBS− and dissociated into a single-cell suspension using Accutase solution (8 min, 37°C). The resulting single cell suspension was diluted in PBS−, centrifuged at 200 g for 5 min, and resuspended in MACS buffer (PBS−, 0.5% FBS, 2 mM EDTA, 10 μM Y-27632). Magnetic-activated cell sorting (MACS) was carried out per manufacturer's instructions (Miltenyi Biotec), i.e. cells were filtered through a 30-μm mesh-filter (Miltenyi Biotec), centrifuged and counted, before staining with mouse anti-CD49a, phycoerythrin (PE)-conjugated antibody (BD Biosciences, #559596; 1:50) for 30 mins in 4°C with constant agitation. After washing with MACS buffer, cells were resuspended in 300 µl of sorting buffer per 10^7^ cells and anti-PE MicroBeads (Miltenyi Biotec) for 20 min at 4°C with constant agitation. Cells were washed twice and resuspended (up to 10^8^ cells) in 1 ml of MACS buffer prior to magnetic separation using LS columns and the MACS Separator (Miltenyi Biotec). Columns were rinsed with 0.5 ml of MACS buffer prior to loading the cell suspension. After washing the loaded columns three times with MACS buffer, they were removed from the separator and cells were eluted using 1 ml of MACS buffer. The positively selected fractions were reaggregated in Aggrewell^TM^400 plates at a concentration of 1.5×10^6^ cells/ml in CB medium (Table S1) supplemented with 10 µM Y-27632 and 1× Pen/Strep (Gibco). The plates were incubated at 37°C under 5% CO_2_ for 72 h. Later, reaggregated cells were collected into six-well non-treated tissue culture plates (Greiner Bio-One) and placed on an orbital shaker set at 100 rpm. Magnetic sorting for CD19 using MACS followed similar procedures as above, with the exception that dissociated SCβ-cells were directly incubated with anti-CD19 MicroBeads (Miltenyi Biotec) instead of anti-CD19 primary antibody.

Magnetic sorting for CD19 by using the EasySep™ Human CD19 Positive Selection Kit II (StemCell Technologies Inc.) involved the same dissociation procedure as above. Cells (10^8^ cells/ml) were resuspended in a 5 ml Falcon round-bottom polystyrene tube (Fisher Scientific). Cells were mixed and incubated with 100 μl/ml EasySep™ Human CD19 Positive Selection Cocktail II and 100 μl/ml of Dextran RapidSpheres™ for 3 min respectively. Tubes were topped up to 2.5 ml and placed it in the EasySep magnet for 3 min before pouring off negatively selected cells by inverting the magnet and tube. This wash procedure was repeated three times.

To calculate the recovery rate following the MACS sorting process, cell samples were analyzed and sorted in parallel using flow cytometry and MACS, respectively. The recovery rate was calculated using: Recovery rate=[number of cells collected in the MACS tube (yield)] / [CD19+ percentage from flow cytometry×total number of cells in the flow cytometry sample (expected yield)]. An automated cell counter (DeNovix Inc) was used to determine the number of cells collected in the MACS tube.

### Flow cytometry

Day (d)25-29 immature SCβ-cell clusters were washed twice with PBS− and dissociated into a single-cell suspension using Accutase solution (8 min, 37°C). The single cell suspension was diluted in PBS−, centrifuged at 200 ***g*** for 5 min, resuspended and filtered through a 40-μm mesh-filter FACS tube. Cells were stained with mouse anti-human CD19 antibody (Miltenyi Biotec, cat# 130-113-730; 1:50) or mouse anti-human connecting (C)-peptide antibody (BD Pharmingen, #565831; 1:100) in the dark for 25 min. Cells were analyzed on a BD LSR II or Fortessa [British Columbia Children's Hospital Research Institute (BCCHRI) Flow Cytometry Core Facility] and data were processed using FlowJo software.

### Live cell imaging

Unsorted, d25 immature SCβ-cell clusters were collected and washed twice with PBS−. Clusters were stained with 75 nM of LysoTracker Blue DND-22 (Invitrogen) in CB medium for 30 min. Clusters were co-stained with CellMask Green (Invitrogen; 1:1000) for 5 min before imaging on glass bottom culture plates (MatTek, P35G-1.5-14-C). Images were taken with a 20× oil objective on the Leica TCS SP8 confocal system. For live immunofluorescence imaging, a Keyence BZ-X800 fluorescence microscope (Keyence, Osaka, Japan) was used with 10× to 40× objectives.

### Intracellular staining

Immature d25 SCβ-cell clusters were washed twice with PBS− and dissociated into a single-cell suspension using Accutase solution (8 min, 37°C). The single cell suspension was diluted in PBS−, centrifuged at 200 ***g*** for 5 min, resuspended and filtered through a 40-μm mesh-filter FACS tube. To stain for surface CD19 expression, cells were incubated with fixable viability dye eF780 (Invitrogen; 1:1000) and mouse anti-human CD19 antibody (1:50) for 25 min. Cells were fixed overnight at 4°C with the IC Fixation Buffer (eBioscience™). The next day, cells were washed twice with 1× Permeabilization Buffer (eBioscience™) before incubating for 30 min in the same buffer with fixable viability dye eF780 (1:1000) and mouse anti-human CD19 antibody (1:50). Stained cells were washed twice in PBS− before flow cytometry analysis.

### Western blotting

500 spheroids were rinsed in PBS and lysed in boiling standard lysis buffer to denature the proteins (Chen et al., 2023; Speckmann et al., 2016). Crude lysates were subjected to sonication (S-4000 with cup horn; Misonix) for 4 min and centrifuged at 10,000 ***g*** for 10 min. Proteins were separated using a precast gel (Bio-Rad Laboratories Mini-PROTEAN TGX) and transferred to a 0.2 µm nitrocellulose membrane (Bio-Rad Laboratories). Membranes were blocked with 5% milk powder in Tris-buffered saline with Tween (TBS-T) and incubated overnight at 4°C with primary antibodies, including mouse anti-human insulin (L6B10; Cell Signaling Technology, #8138 T, 1:10,000), mouse β-actin (Sigma-Aldrich, #A5441, 1:10,000), rabbit anti-human CD-19 (R&D Systems, #MAB11516, 2 µg/ml) and goat anti mScarlet (St John's Laboratory, catalog no. STJ140286, 1:1500 dilution). The membranes were then incubated with horseradish peroxidase–conjugated secondary antibodies (Jackson ImmunoResearch, 1:10,000 dilution) and visualized using ECL Prime reagent (GE Biosciences). Human islets used in this experiment were obtained from the ADI IsletCore under the identifier H2560 (Kin et al., 2024).

### Static glucose stimulated C-peptide secretion assay

50 SCβ-cell clusters were loaded into each well of a 24-well plate (Corning) and incubated in low-glucose (2.8 mM) Krebs–Ringer Bicarbonate HEPES (KRBH) buffer for 1 h in 37°C. Clusters were then transferred to low-glucose and high-glucose (16.7 mM) KRBH buffer for 1 h respectively, and the supernatant was collected. Clusters were then incubated in KRBH buffer containing 16.7 mM glucose and 30 mM KCl (depolarization challenge) for 1 h before the supernatant was collected. Clusters were dispersed using HCl and 70% ethanol to collect total C-peptide content. Supernatant samples were processed using the STELLUX^®^ Chemi Human C-peptide ELISA (ALPCO). Fold-stimulation was calculated using the following formula: Fold-stimulation=C-peptide secretion at high glucose / C-peptide secretion at low glucose.

### Real-time quantitative PCR analyses

For real-time quantitative PCR analyses (RT-qPCR) analyses a total of 150–200 spheroids were collected, and RNA extraction, cDNA synthesis and qPCR were performed as previously described using ABI ViiA7 qPCR system (Sabatini et al., 2013). Data analysis was conducted using the ΔΔCT method, normalized to the housekeeping gene *TBP*. The list of TaqMan primers used is provided in Table S2.

### NanoString nCounter XT gene expression assay

50 clusters were lysed in 100 μl Buffer RLT (Qiagen) containing 1% β-mercaptoethanol (Sigma-Aldrich). A custom nCounter XT Gene Expression Reporter Codeset was used (Dataset 1). Hybridization reactions were set up for nCounter assay as per the manufacturer's (Bruker Spatial Biology) instructions. Briefly, 3 μl of Reporter Codeset, 5 μl of hybridization buffer, 2 μl of Capture Probeset, and 1.5 μl of total cell lysate was incubated at 65°C for 16 h. The next morning, a sample cartridge was loaded with 30 μl of each sample and run on the nCounter SPRINT Profiler (Bruker Spatial Biology). Data were normalized to reference genes *B2M*, *GAPDH*, *GUSB*, *HPRT1*, *POLR2A* and *TBP* and analyzed using the nSolver 4.0 Analysis Software (Bruker Spatial Biology).

### Statistical analysis

Statistical analyses were performed using Prism 8 (GraphPad Software). All data are presented as the mean±s.e.m. Statistically significant differences were assessed using unpaired *t*-tests or one-way ANOVA followed by Tukey's post-hoc tests for multiple comparisons as appropriate. *P*<0.05 was deemed significant.

## Supplementary Material

10.1242/dmm.052376_sup1 Supplementary information

Dataset 1. Codeset details.

## References

[DMM052376C1] Agulnick, A. D., Ambruzs, D. M., Moorman, M. A., Bhoumik, A., Cesario, R. M., Payne, J. K., Kelly, J. R., Haakmeester, C., Srijemac, R., Wilson, A. Z. et al. (2015). Insulin-producing endocrine cells differentiated in vitro from human embryonic stem cells function in macroencapsulation devices in vivo. *Stem Cells Transl. Med.* 4, 1214-1222. 10.5966/sctm.2015-0079 26304037 PMC4572906

[DMM052376C2] Alvarez-Dominguez, J. R., Donaghey, J., Rasouli, N., Kenty, J. H. R., Helman, A., Charlton, J., Straubhaar, J. R., Meissner, A. and Melton, D. A. (2020). Circadian entrainment triggers maturation of human in vitro islets. *Cell Stem Cell* 26, 108-122.e10. 10.1016/j.stem.2019.11.011 31839570

[DMM052376C3] Ameri, J., Borup, R., Prawiro, C., Ramond, C., Schachter, K. A., Scharfmann, R. and Semb, H. (2017). Efficient generation of glucose-responsive beta cells from isolated GP2+ human pancreatic progenitors. *Cell Rep.* 19, 36-49. 10.1016/j.celrep.2017.03.032 28380361

[DMM052376C4] Arcangeli, S., Falcone, L., Camisa, B., De Girardi, F., Biondi, M., Giglio, F., Ciceri, F., Bonini, C., Bondanza, A. and Casucci, M. (2020). Next-generation manufacturing protocols enriching TSCM CAR T cells can overcome disease-specific T cell defects in cancer patients. *Front. Immunol.* 11, 1217. 10.3389/fimmu.2020.01217 32636841 PMC7317024

[DMM052376C5] Augsornworawat, P., Maxwell, K. G., Velazco-Cruz, L. and Millman, J. R. (2020). Single-cell transcriptome profiling reveals β cell maturation in stem cell-derived islets after transplantation. *Cell Rep.* 32, 108067. 10.1016/j.celrep.2020.108067 32846125 PMC7491368

[DMM052376C6] Bagashev, A., Sotillo, E., Anthony Tang, C.-H., Black, K. L., Perazzelli, J., Seeholzer, S. H., Argon, Y., Barrett, D. M., Grupp, S. A., Andrew Hu, C.-C. et al. (2018). CD19 alterations emerging after CD19-directed immunotherapy cause retention of the misfolded protein in the endoplasmic reticulum. *Mol. Cell. Biol.*. 38, e00383-18. 10.1128/MCB.00383-18 30104252 PMC6189457

[DMM052376C7] Balboa, D., Barsby, T., Lithovius, V., Saarimäki-Vire, J., Omar-Hmeadi, M., Dyachok, O., Montaser, H., Lund, P. E., Yang, M., Ibrahim, H. et al. (2022). Functional, metabolic and transcriptional maturation of human pancreatic islets derived from stem cells. *Nat. Biotechnol.* 40, 1042-1055. 10.1038/s41587-022-01219-z 35241836 PMC9287162

[DMM052376C8] Bellin, M. D., Barton, F. B., Heitman, A., Harmon, J. V., Kandaswamy, R., Balamurugan, A. N., Sutherland, D. E. R., Alejandro, R. and Hering, B. J. (2012). Potent induction immunotherapy promotes long-term insulin independence after islet transplantation in type 1 diabetes. *Am. J. Transplant.* 12, 1576-1583. 10.1111/j.1600-6143.2011.03977.x 22494609 PMC3390261

[DMM052376C9] Bindels, D. S., Haarbosch, L., Van Weeren, L., Postma, M., Wiese, K. E., Mastop, M., Aumonier, S., Gotthard, G., Royant, A., Hink, M. A. et al. (2017). MScarlet: A bright monomeric red fluorescent protein for cellular imaging. *Nat. Methods* 14, 53-56. 10.1038/nmeth.4074 27869816

[DMM052376C10] Calderari, S., Gangnerau, M. N., Thibault, M., Meile, M. J., Kassis, N., Alvarez, C., Portha, B. and Serradas, P. (2007). Defective IGF2 and IGF1R protein production in embryonic pancreas precedes beta cell mass anomaly in the Goto-Kakizaki rat model of type 2 diabetes. *Diabetologia* 50, 1463-1471. 10.1007/s00125-007-0676-2 17476475

[DMM052376C11] Chen, Y. C., Taylor, A. J., Fulcher, J. M., Swensen, A. C., Dai, X. Q., Komba, M., Wrightson, K. L. C., Fok, K., Patterson, A. E., Geltink, R. I. K. et al. (2023). Deletion of carboxypeptidase E in β-cells disrupts proinsulin processing but does not lead to spontaneous development of diabetes in mice. *Diabetes* 72, 1277-1288. 10.2337/db22-0945 37364047 PMC10450824

[DMM052376C12] Cogger, K. F., Sinha, A., Sarangi, F., McGaugh, E. C., Saunders, D., Dorrell, C., Mejia-Guerrero, S., Aghazadeh, Y., Rourke, J. L., Screaton, R. A. et al. (2017). Glycoprotein 2 is a specific cell surface marker of human pancreatic progenitors. *Nat. Commun.* 8, 331. 10.1038/s41467-017-00561-0 28835709 PMC5569081

[DMM052376C13] D'Amour, K. A., Bang, A. G., Eliazer, S., Kelly, O. G., Agulnick, A. D., Smart, N. G., Moorman, M. A., Kroon, E., Carpenter, M. K. and Baetge, E. E. (2006). Production of pancreatic hormone-expressing endocrine cells from human embryonic stem cells. *Nat. Biotechnol.* 24, 1392-1401. 10.1038/nbt1259 17053790

[DMM052376C14] Fong, C. Y., Peh, G. S. L., Gauthaman, K. and Bongso, A. (2009). Separation of SSEA-4 and TRA-1-60 labelled undifferentiated human embryonic stem cells from a heterogeneous cell population using magnetic-activated cell sorting (MACS) and fluorescence-activated cell sorting (FACS). *Stem Cell Rev. Rep.* 5, 72-80. 10.1007/s12015-009-9054-4 19184635

[DMM052376C15] Gadella, T. W. J., van Weeren, L., Stouthamer, J., Hink, M. A., Wolters, A. H. G., Giepmans, B. N. G., Aumonier, S., Dupuy, J. and Royant, A. (2023). mScarlet3: a brilliant and fast-maturing red fluorescent protein. *Nat. Methods* 20, 541-545. 10.1038/s41592-023-01809-y 36973546

[DMM052376C16] Groves, C. J., Carrell, J., Grady, R., Rajan, B., Morehouse, C. A., Halpin, R., Wang, J., Wu, J., Shrestha, Y., Rayanki, R. et al. (2018). CD19-positive antibody-secreting cells provide immune memory. *Blood Adv.* 2, 3163-3176. 10.1182/bloodadvances.2017015172 30478153 PMC6258909

[DMM052376C17] Heard, A., Landmann, J. H., Hansen, A. R., Papadopolou, A., Hsu, Y. S., Selli, M. E., Warrington, J. M., Lattin, J., Chang, J., Ha, H. et al. (2022). Antigen glycosylation regulates efficacy of CAR T cells targeting CD19. *Nat. Commun.* 13, 3367. 10.1038/s41467-022-31035-7 35690611 PMC9188573

[DMM052376C18] Hiyoshi, H., Sakuma, K., Tsubooka-Yamazoe, N., Asano, S., Mochida, T., Yamaura, J., Konagaya, S., Fujii, R., Matsumoto, H., Ito, R. et al. (2022). Characterization and reduction of non-endocrine cells accompanying islet-like endocrine cells differentiated from human iPSC. *Sci. Rep.* 12, 4740. 10.1038/s41598-022-08753-5 35304548 PMC8933508

[DMM052376C19] Hockemeyer, D., Wang, H., Kiani, S., Lai, C. S., Gao, Q., Cassady, J. P., Cost, G. J., Zhang, L., Santiago, Y., Miller, J. C. et al. (2011). Genetic engineering of human pluripotent cells using TALE nucleases. *Nat. Biotechnol.* 29, 731-734. 10.1038/nbt.1927 21738127 PMC3152587

[DMM052376C20] Hogrebe, N. J., Augsornworawat, P., Maxwell, K. G., Velazco-Cruz, L. and Millman, J. R. (2020). Targeting the cytoskeleton to direct pancreatic differentiation of human pluripotent stem cells. *Nat. Biotechnol.* 38, 460-470. 10.1038/s41587-020-0430-6 32094658 PMC7274216

[DMM052376C21] Hughes, A., Mohanasundaram, D., Kireta, S., Jessup, C. F., Drogemuller, C. J. and Coates, P. T. H. (2013). Insulin-like growth factor-II (IGF-II) prevents proinflammatory cytokine-induced apoptosis and significantly improves islet survival after transplantation. *Transplantation* 95, 671-678. 10.1097/TP.0b013e31827fa453 23364485

[DMM052376C22] Kelly, O. G., Chan, M. Y., Martinson, L. A., Kadoya, K., Ostertag, T. M., Ross, K. G., Richardson, M., Carpenter, M. K., D'Amour, K. A., Kroon, E. et al. (2011). Cell-surface markers for the isolation of pancreatic cell types derived from human embryonic stem cells. *Nat. Biotechnol.* 29, 750-756. 10.1038/nbt.1931 21804561

[DMM052376C23] Kim, J. H., Lee, S. R., Li, L. H., Park, H. J., Park, J. H., Lee, K. Y., Kim, M. K., Shin, B. A. and Choi, S. Y. (2011). High cleavage efficiency of a 2A peptide derived from porcine teschovirus-1 in human cell lines, zebrafish and mice. *PLoS ONE* 6, e18556. 10.1371/journal.pone.0018556 21602908 PMC3084703

[DMM052376C24] Kin, T., O'Gorman, D., Zhai, W., Moriarty, J., Park, K., Ganguly, A., Rosichuk, S. and Shapiro, A. M. J. (2024). Contribution of a single islet transplant program to basic researchers in North America, Europe, and ASIA through distributing human islets. *OBM Transpl.* 8, 212. 10.21926/obm.transplant.2402212

[DMM052376C25] Korytnikov, R. and Nostro, M. C. (2016). Generation of polyhormonal and multipotent pancreatic progenitor lineages from human pluripotent stem cells. *Methods* 101, 56-64. 10.1016/j.ymeth.2015.10.017 26515645

[DMM052376C26] Krentz, N. A. J., Nian, C. and Lynn, F. C. (2014). TALEN/CRISPR-mediated eGFP knock-in add-on at the OCT4 locus does not impact differentiation of human embryonic stem cells towards endoderm. *PLoS ONE* 9, e114275. 10.1371/journal.pone.0114275 25474420 PMC4256397

[DMM052376C27] Kroon, E., Martinson, L. A., Kadoya, K., Bang, A. G., Kelly, O. G., Eliazer, S., Young, H., Richardson, M., Smart, N. G., Cunningham, J. et al. (2008). Pancreatic endoderm derived from human embryonic stem cells generates glucose-responsive insulin-secreting cells in vivo. *Nat. Biotechnol.* 26, 443-452. 10.1038/nbt1393 18288110

[DMM052376C28] Liu, Z., Chen, O., Wall, J. B. J., Zheng, M., Zhou, Y., Wang, L., Ruth Vaseghi, H., Qian, L. and Liu, J. (2017). Systematic comparison of 2A peptides for cloning multi-genes in a polycistronic vector. *Sci. Rep.* 7, 2193. 10.1038/s41598-017-02460-2 28526819 PMC5438344

[DMM052376C29] Mahaddalkar, P. U., Scheibner, K., Pfluger, S., Ansarullah, Sterr, M., Beckenbauer, J., Irmler, M., Beckers, J., Knöbel, S. and Lickert, H. (2020). Generation of pancreatic β cells from CD177+ anterior definitive endoderm. *Nat. Biotechnol.* 38, 1061-1072. 10.1038/s41587-020-0492-5 32341565

[DMM052376C30] Mar, S., Filatov, E., Sasaki, S., Mojibian, M., Zhang, D., Yang, A., Nian, C. and Lynn, F. C. (2025). Tracking insulin- and glucagon-expressing cells in vitro and in vivo using a double-reporter human embryonic stem cell line. *Diabetes* 74, 188-198. 10.2337/db24-0756 39561351 PMC11755683

[DMM052376C31] Micallef, S. J., Li, X., Schiesser, J. V., Hirst, C. E., Yu, Q. C., Lim, S. M., Nostro, M. C., Elliott, D. A., Sarangi, F., Harrison, L. C. et al. (2012). INSGFP/w human embryonic stem cells facilitate isolation of in vitro derived insulin-producing cells. *Diabetologia* 55, 694-706. 10.1007/s00125-011-2379-y 22120512 PMC3268987

[DMM052376C32] Monk, D., Sanches, R., Arnaud, P., Apostolidou, S., Hills, F. A., Abu-Amero, S., Murrell, A., Friess, H., Reik, W., Stanier, P. et al. (2006). Imprinting of IGF2 P0 transcript and novel alternatively spliced INS-IGF2 isoforms show differences between mouse and human. *Hum. Mol. Genet.* 15, 1259-1269. 10.1093/hmg/ddl041 16531418

[DMM052376C33] Nair, G. G., Liu, J. S., Russ, H. A., Tran, S., Saxton, M. S., Chen, R., Juang, C., Li, M. L., Nguyen, V. Q., Giacometti, S. et al. (2019). Recapitulating endocrine cell clustering in culture promotes maturation of human stem-cell-derived β cells. *Nat. Cell Biol.* 21, 263-274. 10.1038/s41556-018-0271-4 30710150 PMC6746427

[DMM052376C34] Novakovsky, G., Sasaki, S., Fornes, O., Omur, M. E., Huang, H., Bayly, C. L., Zhang, D., Lim, N., Cherkasov, A., Pavlidis, P. et al. (2023). In silico discovery of small molecules for efficient stem cell differentiation into definitive endoderm. *Stem Cell Rep.* 18, 765-781. 10.1016/j.stemcr.2023.01.008 PMC10031281 36801003

[DMM052376C35] Pagliuca, F. W., Millman, J. R., Gürtler, M., Segel, M., Van Dervort, A., Ryu, J. H., Peterson, Q. P., Greiner, D. and Melton, D. A. (2014). Generation of functional human pancreatic β cells in vitro. *Cell* 159, 428-439. 10.1016/j.cell.2014.09.040 25303535 PMC4617632

[DMM052376C36] Parent, A. V., Ashe, S., Nair, G. G., Li, M. L., Chavez, J., Liu, J. S., Zhong, Y., Streeter, P. R. and Hebrok, M. (2022). Development of a scalable method to isolate subsets of stem cell-derived pancreatic islet cells. *Stem Cell Rep.* 17, 979-992. 10.1016/j.stemcr.2022.02.001 PMC9023773 35245441

[DMM052376C37] Rezania, A., Bruin, J. E., Arora, P., Rubin, A., Batushansky, I., Asadi, A., Dwyer, S. O., Quiskamp, N., Mojibian, M., Albrecht, T. et al. (2014). Reversal of diabetes with insulin-producing cells derived in vitro from human pluripotent stem cells. *Nat. Biotechnol.* 32, 1121-1133. 10.1038/nbt.3033 25211370

[DMM052376C38] Robert, T., De Mesmaeker, I., Stangé, G. M., Suenens, K. G., Ling, Z., Kroon, E. J. and Pipeleers, D. G. (2018). Functional beta cell mass from device-encapsulated hESC-derived pancreatic endoderm achieving metabolic control. *Stem Cell Rep.* 10, 739-750. 10.1016/j.stemcr.2018.01.040 PMC5918665 29503087

[DMM052376C39] Russ, H. A., Parent, A. V., Ringler, J. J., Hennings, T. G., Nair, G. G., Shveygert, M., Guo, T., Puri, S., Haataja, L., Cirulli, V. et al. (2015). Controlled induction of human pancreatic progenitors produces functional beta–like cells in vitro . *EMBO J.* 34, 1759-1772. 10.15252/embj.201591058 25908839 PMC4516429

[DMM052376C40] Sabatini, P. V., Krentz, N. A. J., Zarrouki, B., Westwell-Roper, C. Y., Nian, C., Uy, R. A., Shapiro, A. M. J., Poitout, V. and Lynn, F. C. (2013). Npas4 is a novel activity-regulated cytoprotective factor in pancreatic β-cells. *Diabetes* 62, 2808-2820. 10.2337/db12-1527 23656887 PMC3717850

[DMM052376C41] Sandovici, I., Hammerle, C. M., Virtue, S., Vivas-Garcia, Y., Izquierdo-Lahuerta, A., Ozanne, S. E., Vidal-Puig, A., Medina-Gómez, G. and Constância, M. (2021). Autocrine IGF2 programmes β-cell plasticity under conditions of increased metabolic demand. *Sci. Rep.* 11, 7717. 10.1038/s41598-021-87292-x 33833312 PMC8032793

[DMM052376C42] Shapiro, A. M. J. (2012). Islet transplantation in type 1 diabetes: Ongoing challenges, refined procedures, and long-term outcome. *Rev. Diabetic Stud.* 9, 385-406. 10.1900/RDS.2012.9.385 23804275 PMC3740705

[DMM052376C43] Shapiro, A. M. J. and Verhoeff, K. (2023). A spectacular year for islet and stem cell transplantation. *Nat. Rev. Endocrinol.* 19, 68-69. 10.1038/s41574-022-00790-4 36539606

[DMM052376C44] Shapiro, A. M. J., Pokrywczynska, M. and Ricordi, C. (2017). Clinical pancreatic islet transplantation. *Nat. Rev. Endocrinol.* 13, 268-277. 10.1038/nrendo.2016.178 27834384

[DMM052376C45] Sneddon, J. B., Tang, Q., Stock, P., Bluestone, J. A., Roy, S., Desai, T. and Hebrok, M. (2018). Stem cell therapies for treating diabetes: progress and remaining challenges. *Cell Stem Cell* 22, 810-823. 10.1016/j.stem.2018.05.016 29859172 PMC6007036

[DMM052376C46] Speckmann, T., Sabatini, P. V., Nian, C., Smith, R. G. and Lynn, F. C. (2016). Npas4 transcription factor expression is regulated by calcium signaling pathways and prevents tacrolimus-induced cytotoxicity in pancreatic beta cells. *J. Biol. Chem.* 291, 2682-2695. 10.1074/jbc.M115.704098 26663079 PMC4742737

[DMM052376C47] Tedder, T. F., Inaoki, M. and Sato, S. (1997). The CD19-CD21 complex regulates signal transduction thresholds governing humoral immunity and autoimmunity. *Immunity* 6, 107-118. 10.1016/S1074-7613(00)80418-5 9047233

[DMM052376C48] Uhlén, M., Fagerberg, L., Hallström, B. M., Lindskog, C., Oksvold, P., Mardinoglu, A., Sivertsson, Å., Kampf, C., Sjöstedt, E., Asplund, A. et al. (2015). Tissue-based map of the human proteome. *Science* 347, 1260419. 10.1126/science.1260419 25613900

[DMM052376C49] Van Rosmalen, M., Krom, M. and Merkx, M. (2017). Tuning the flexibility of glycine-serine linkers to allow rational design of multidomain proteins. *Biochemistry* 56, 6565-6574. 10.1021/acs.biochem.7b00902 29168376 PMC6150656

[DMM052376C50] Velazco-Cruz, L., Song, J., Maxwell, K. G., Goedegebuure, M. M., Augsornworawat, P., Hogrebe, N. J. and Millman, J. R. (2019). Acquisition of dynamic function in human stem cell-derived β cells. *Stem Cell Rep.* 12, 351-365. 10.1016/j.stemcr.2018.12.012 PMC6372986 30661993

[DMM052376C51] Veres, A., Faust, A. L., Bushnell, H. L., Engquist, E. N., Kenty, J. H. R., Harb, G., Poh, Y. C., Sintov, E., Gürtler, M., Pagliuca, F. W. et al. (2019). Charting cellular identity during human in vitro β-cell differentiation. *Nature* 569, 368-373. 10.1038/s41586-019-1168-5 31068696 PMC6903417

[DMM052376C52] Wang, K., Wei, G. and Liu, D. (2012). CD19: a biomarker for B cell development, lymphoma diagnosis and therapy. *Exp Hematol. Oncol.* 1, 1-7. 10.1186/2162-3619-1-36 23210908 PMC3520838

[DMM052376C53] Zhang, W., Jordan, K. R., Schulte, B. and Purev, E. (2018). Characterization of clinical grade CD19 chimeric antigen receptor T cells produced using automated CliniMACS Prodigy system. *Drug Des. Devel. Ther.* 12, 3343-3356. 10.2147/DDDT.S175113 PMC6181073 30323566

[DMM052376C54] Zhu, F., Shah, N., Xu, H., Schneider, D., Orentas, R., Dropulic, B., Hari, P. and Keever-Taylor, C. A. (2018). Closed-system manufacturing of CD19 and dual-targeted CD20/19 chimeric antigen receptor T cells using the CliniMACS Prodigy device at an academic medical center. *Cytotherapy* 20, 394-406. 10.1016/j.jcyt.2017.09.005 29287970

