# Supplementary material for: Truncated CD19 as a selection marker for the isolation of stem cell-derived β-cells
Source: Dis Model Mech. 2026 Jan 5;19(1):dmm052376. doi: 10.1242/dmm.052376 (PMC12817333; doi:10.1242/dmm.052376)
Supplement: Supplementary information [file dmm-19-052376-s1.pdf]

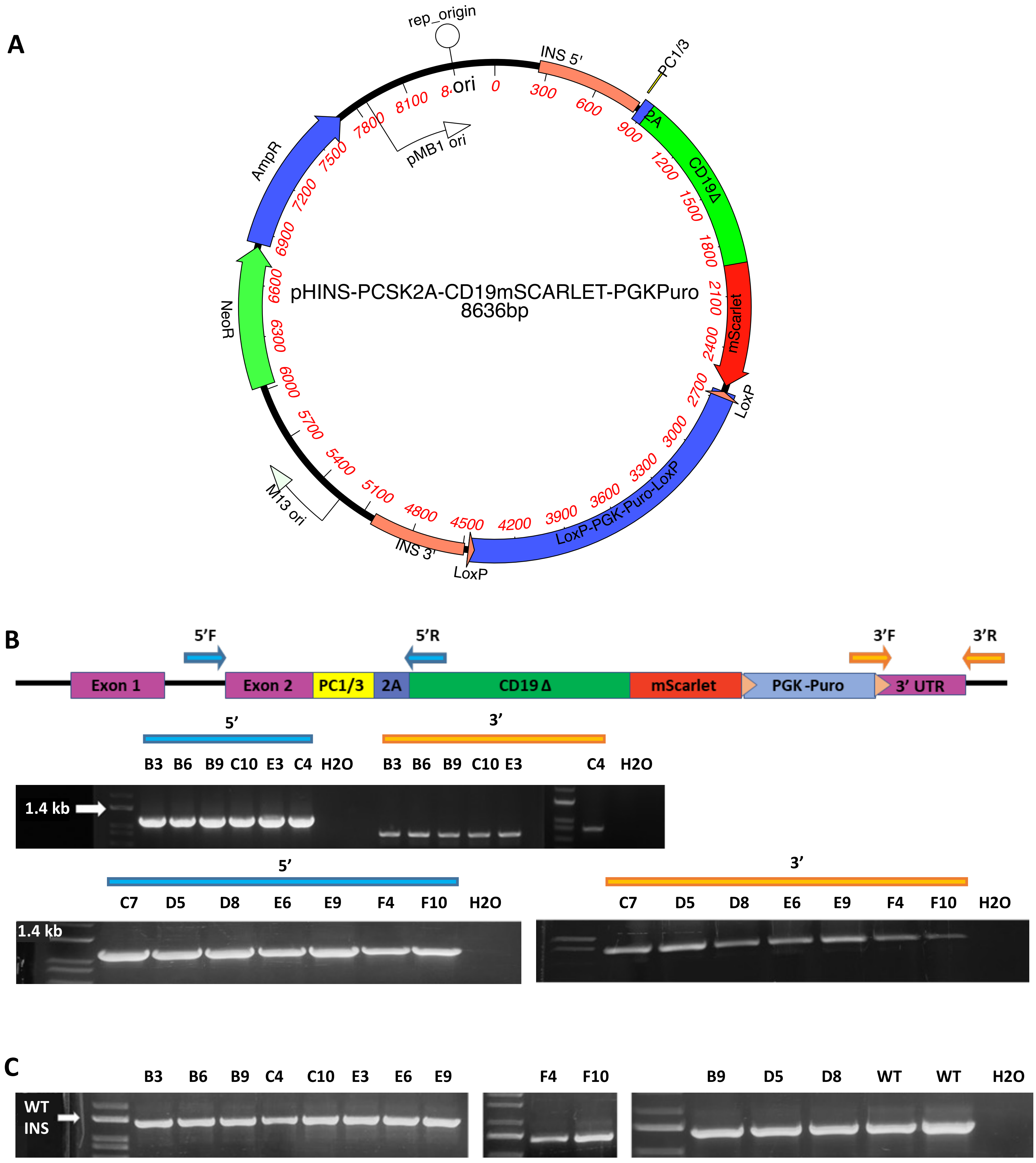

Fig. S1. Cloning and selection strategy. (A) Schematic representation of the complete DNA donor vector, featuring 800bp of INS homology arms and the human codon-optimized version of PCSK1/3-P2A-CD19Δ-mScarlet. (B) Genotyping PCR for 3' and 5' arms of insertion. Regions where genotyping PCR primer pairs bind at 5' and 3' highlighted in blue arrows. (C) Endogenous INS allele assayed with primers 5'F and 3'R for thirteen representative knock-in add-on hESC clones.

Disease Models & Mechanisms • Supplementary information

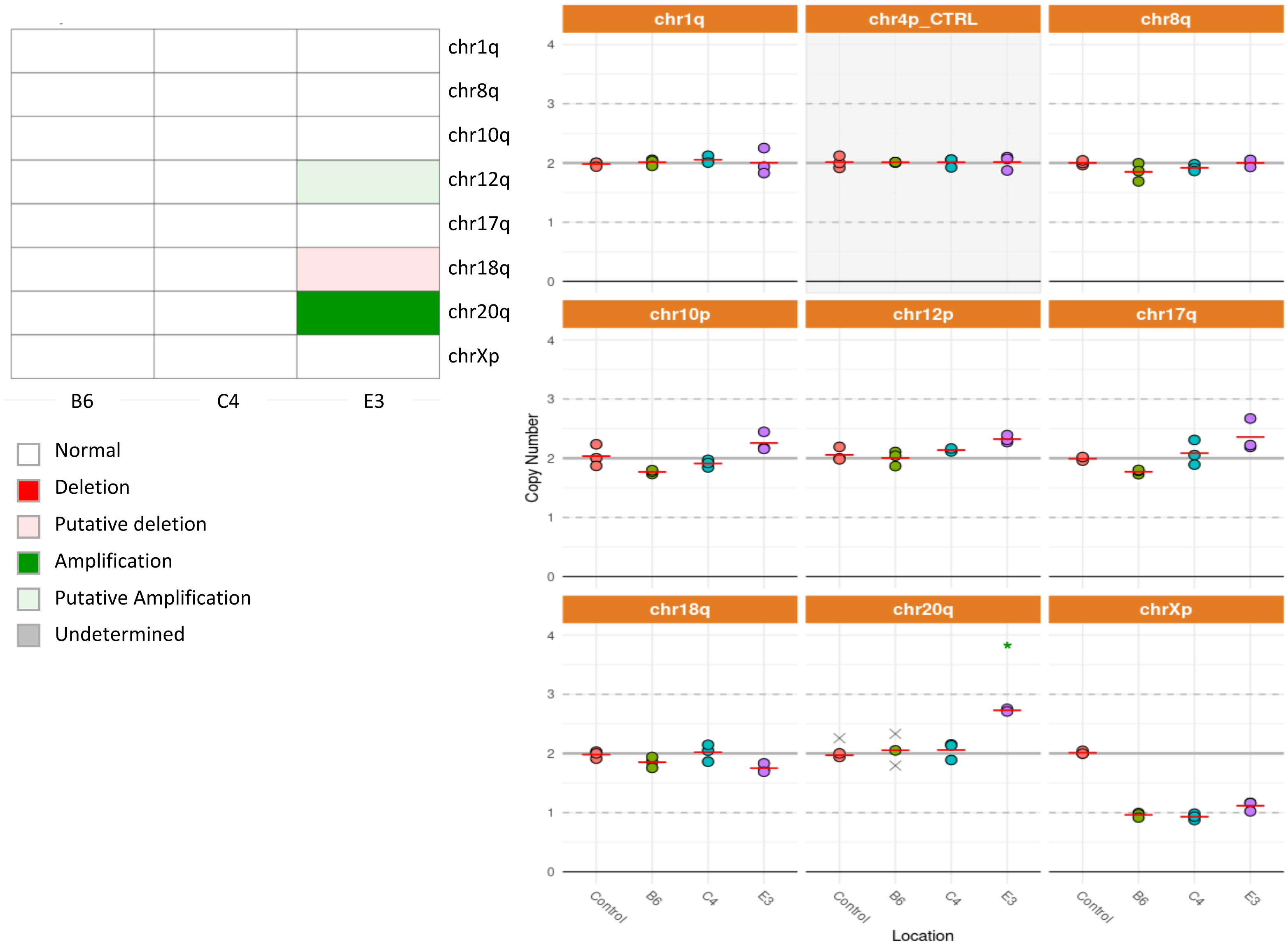

Fig. S2. qPCR-based profiling of genomic abnormalities in INS-2A-CD19-mScarlet cell clonesclones 4 and E3. N=3 technical replicates. The probes represent eight common karyotypic abnormalities that have been reported to arise in hESC and hiPSC: chr 1q, chr 8q, chr 10p, chr 12p, chr 17q, chr 18q, chr 20q or chr Xp. Sample-probe mixes were analyzed on a ViAATM7 PCR System. Results were normalized to the copy number of a control region in chr 4p and analyzed using the ΔΔCt method.

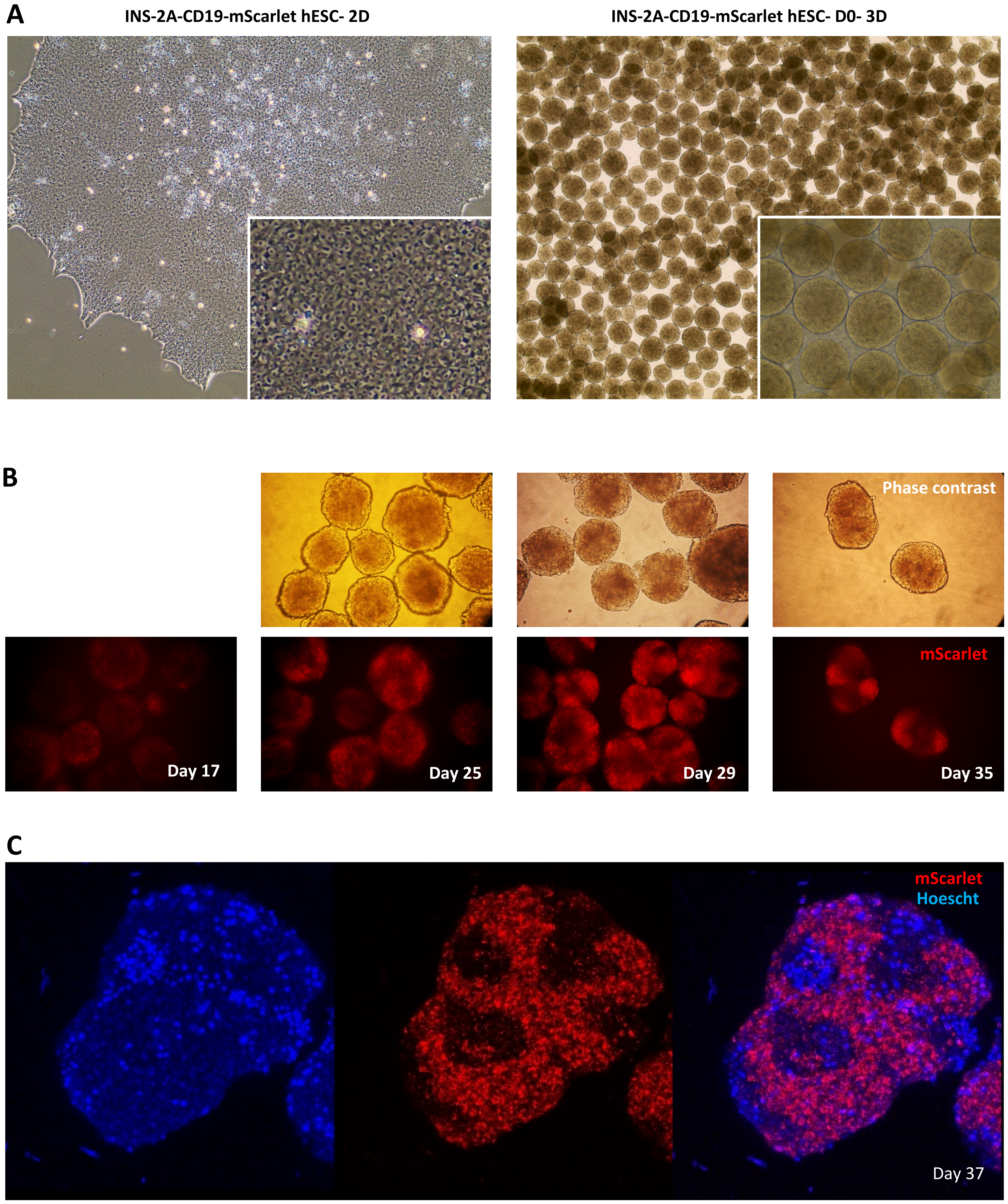

Fig. S3. INS-2A-CD19-mScarlet hESC line during culturing and differentiation. (A) INS-2A-CD19-mScarlet hESC line on day 4 (d4) cultured on a Cultrex-coated 10 cm plate (left) and on day 0 (d0), one day after seeding into 6-well low-adherent plates at a density of 1000 cells per well. (B) Morphology and mScarlet expression profile of spheroids at differentiation daysdays 17, 25, 2 nd 35. (C) Live confocal imaging of unsorted stage 6 (d37) immature β-like clusters expressing INS-2A-CD19-mScarlet (red). Scale bars:clust m.

Disease Models & Mechanisms • Supplementary information

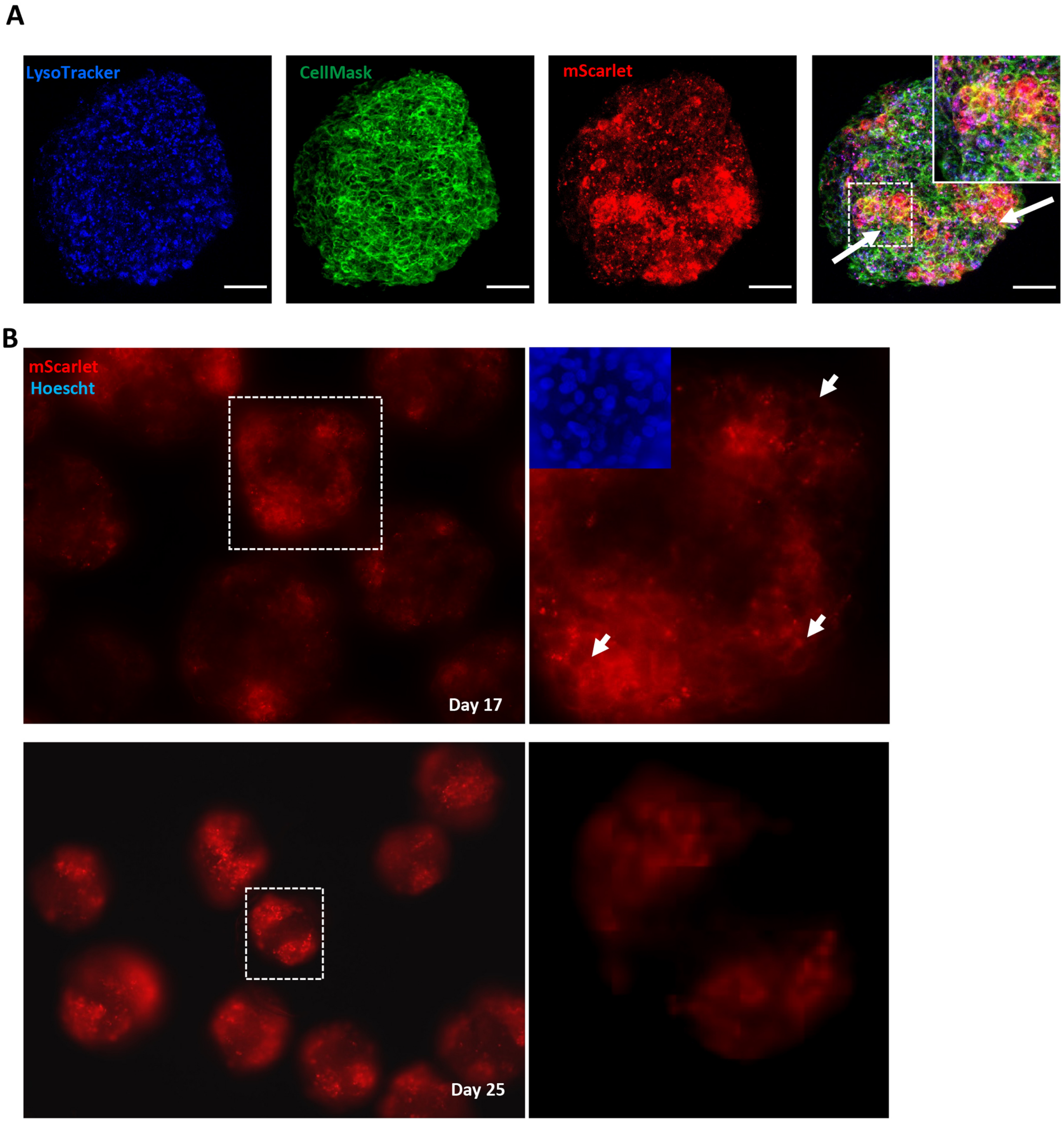

Fig. S4. Surface expression of CD19-mScarlet in the INS-2A-CD19-mScarlet hESC line during differentiation. (A) Live confocal imaging of unsorted stage 6, d25 immature β-like clusters expressing INS-2A-CD19Δ-mScarlet (red). Clusters were stained with LysoTracker (blue) and CellMask (green). Scale bars represent 50 µm. (B) Live immunofluorescence analysis of unsorted d17 and d25 immature β-like clusters expressing INS-2A-CD19Δ-mScarlet (red). Clusters were stained for Hoescht (blue).

Disease Models & Mechanisms • Supplementary information

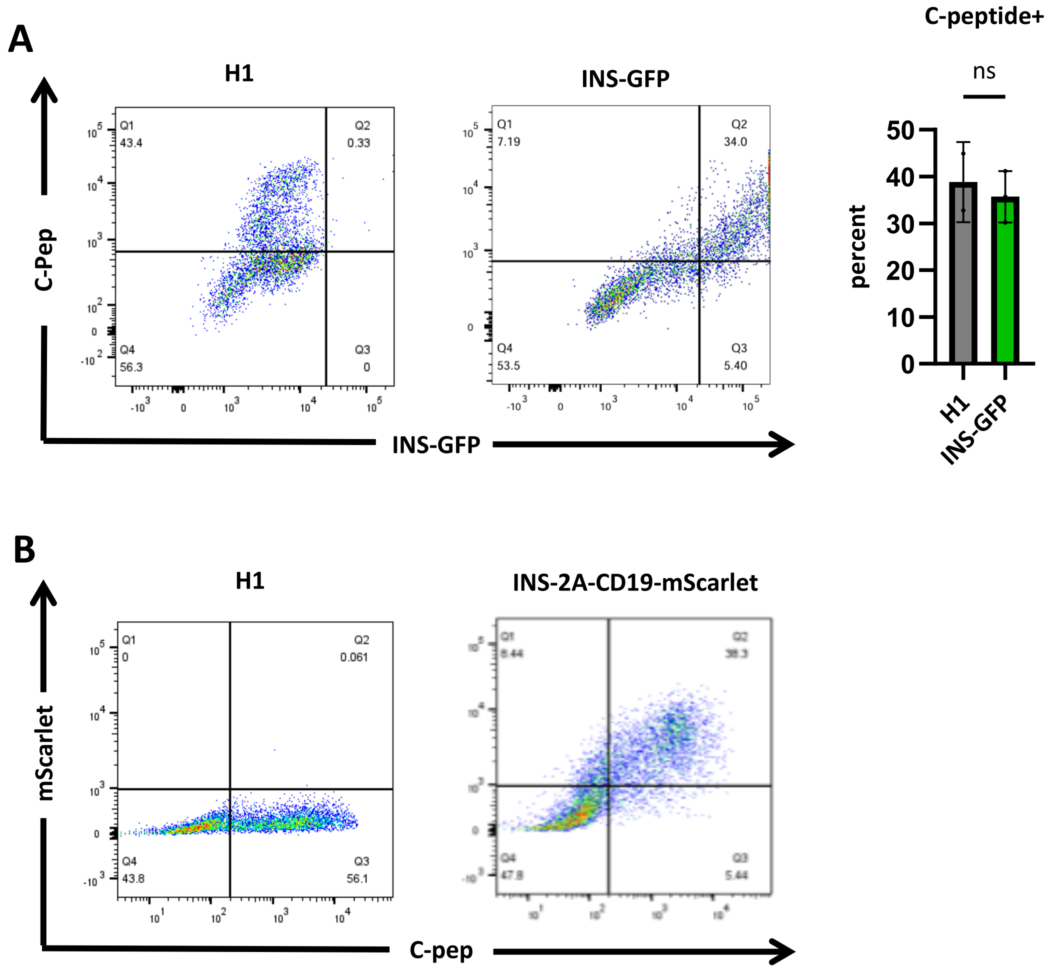

Fig. S5. Differentiation efficiency in transgenic hESC lines. (A) Representative flow cytometry analysis of c-peptide and INS-GFP expression in Stage 6, Day 21 cells derived from parental H1 and INS-EGFP #26 cell lines. Data are based on N=2 independent biological replicates for H1 and N=3 independent biological replicates for INS-GFP. Statistical significance was determined using an unpaired t-test. (B) Representative flow cytometry analysis of c-peptide and mScarlet expression in Stage 6, Day 29 cells derived from parental H1 and INS-2A-CD19-mScarlet hESC lines. Data are based on N=3 independent biological replicates. These data provide an alternative representation of results shown in Figs 1C and 2E.

A

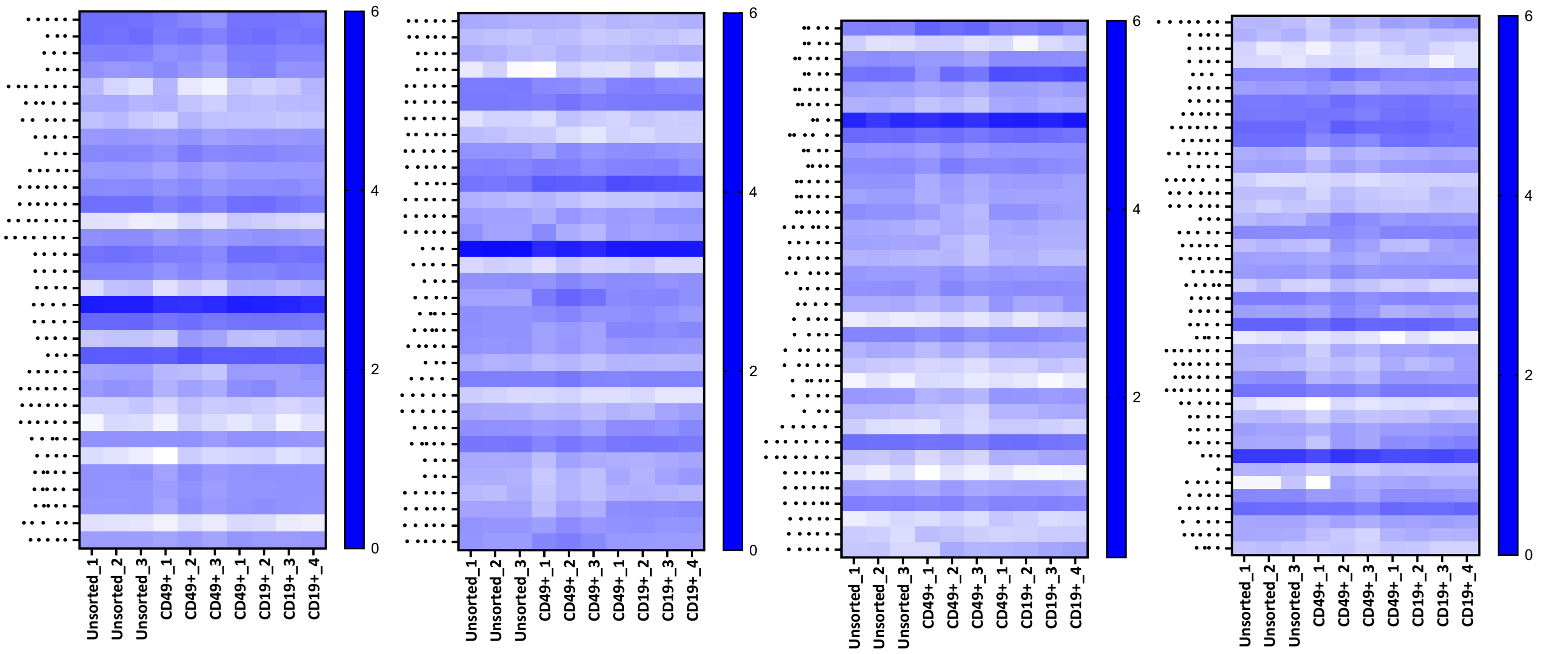

B

Gene annotations	No. of genes in category	No. of DEGs in Un vs Sorted in category	No. of DEGs in CD49 vs CD19
alpha cell	4	1	0
beta cell	14	4	4
beta cell/alpha cell	6	1	0
delta cell	2	1	0
Endocrine	20	10	5
endocrine & exocrine	4	2	0
endoderm cell	6	1	1
endothelial cell	4	2	0
epsilon cells	1	1	0
exocrine/ductal	17	1	1
mesoderm cell	5	2	1
other endodermal cell types	10	4	3
other genes	48	8	3
pancreatic immune cell	2	2	1
PP cell	1	1	0
stem cell	5	0	0
**Total**	**149**	**41**	**19**

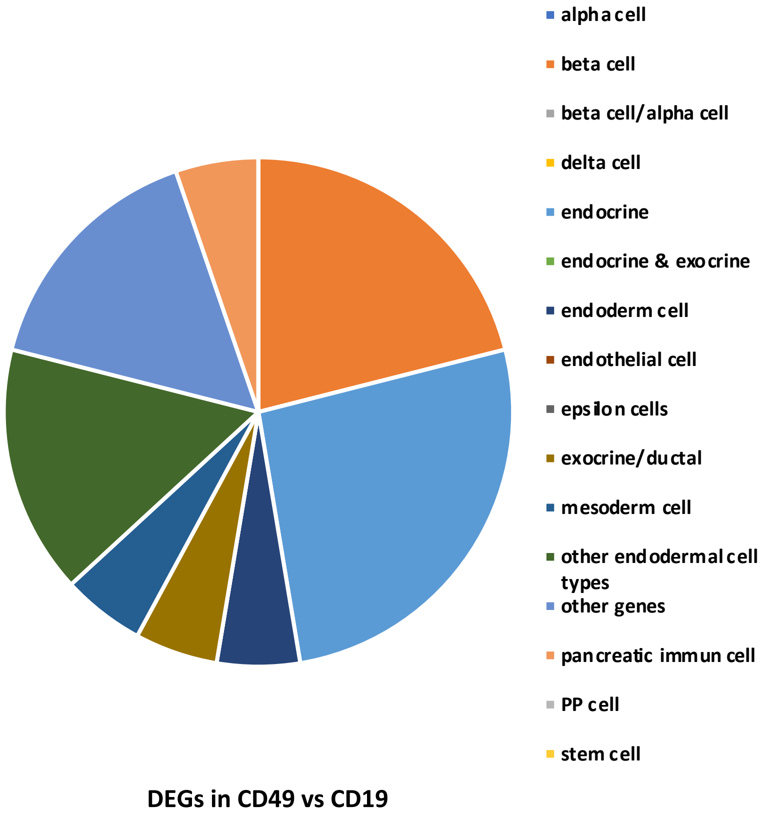

Fig. S6. Gene expression analysis of unsorted, CD49a sorted and CD19 sorted SCβ-cells with Nanostring. (A) Heatmap showing the expression counts of 149 evaluated genes, normalized to the expression levels of reference genes. (B) The table provides the annotations for these 149 genes, categorizing them into 16 groups. It also presents the number of genes that showed significant expression changes after sorting based on CD49 and CD19, as well as those that exhibited significant differences between CD49- and CD19-sorted cells. The graph illustrates the percentage of significantly altered genes between the two sorting methods.

Disease Models & Mechanisms • Supplementary information

Table S1. Media composition for all stages of differentiation.

Time point	Stage	Media	Additives
d(-1)	Aggregation	StemFlex or mTESR+	
d0	1	RB+0.2%FBS+1:5000 ITS	100ng/ml Activin A; 3µM CHIR99021; 1µM JNJ
d1		RB+0.2%FBS+1:2000 ITS	100ng/ml Activin A; 1 µM JNJ
d2	2	RB+0.2%FBS+1:1000 ITS	25ng/ml KGF
d3		RB+0.4%FBS+1:1000 ITS	25ng/ml KGF
d4		RB+0.4%FBS+1:1000 ITS	25ng/ml KGF
d5	3	DB*+1:100 B27	3nM TTNPB; 250nM SANT-1; 250nM PdBU; 250nM LDN; 0.5mM 2-P-L-AA
d6		DB*+1:100 B27	3nM TTNPB; 0.5mM 2-P-L-AA
d8	4	DB*+1:100 B27	50ng/ml EGF; 50ng/ml KGF; 0.5mM 2-P-LAA
d9		DB*+1:100 B27	50ng/ml EGF; 50ng/ml KGF; 0.5mM 2-P-LAA
d10-d14	induction	DB2	10µM Alk5i (RepSox); 2µM T3; 0.5µM LDN; 1µM Xxi
d15-21	maturation	CB	10µM Alk5i (RepSox); 2µM T3; 0.155mM 2-P-L-AA
d21-27	maturation 2	CB	2µM T3; 0.155mM 2-P-LAA
d27+	Balboa	CB	10nM T3; 1:2000 Trace A; 1:2000 Trace B; 1:2000 Lipid Conc; 0.5µM ZM447439
RB	RPMI 1640 (Hyclone SH30096) + 1x Glutamax + 1/200 Pen/Strep		
DB*	DMEM (high glucose; Hyclone SH 30081 or Gibco 11960) + 1x Glutamax + 1x NEAA + 1mM Sodium Pyruvate + 1/200 Pen/Strep		
DB2	DMEM + 1x Glutamax + 1x NEAA + 1mM Pyruvate + 1% BSA (Fatty Acid Free, Fraction V) + 1x ITS + 1/200 Pen/Strep + 10 µg/ml Heparin + 2µM NAC + 10 µM Zn Sulphate + 1.75 µl ß-ME/500ml		
CB	CMRL (5.6mM Glucose; eg CellGro 15-110-CV) + 1x Glutamax + 1x NEAA + 1mM Pyruvate + 1% BSA (Fatty Acid Free, Fraction V) + 1x ITS + 1/200 Pen/Strep + 1x HEPES + 10 µg/ml Heparin + 2µM NAC + 10 µM Zn Sulphate + 1.75 µl ß-ME/500ml -> pH to 7.4 and filter		

Table S2. TAQMAN probes and primers.

Target Gene	Probe	Primer 1	Primer 2
MAFA	TTG TAC AGG TCC CGC TCT TTG GC	GAG AAG TGC CAA CTC CAG AG	GCC AGC TTC TCG TAT TTC TCC
PCSK1	TCC CGA AGA GGA GAC CTT CAT GTC A	CTA TCA AGT CCC TGG AGC ATG	TGT ATC CCG TTC TCT TTC AGC
PDX1	CGC TTG TTC TCC TCC GGC TCC	TGA AGT CTA CCA AAG CTC ACG	GGA ACT CCT TCT CCA GCT CTA
ISL1	CAG TGG AAT TAG AGC CCG GTC CT	GGT GTA TCT GGA AGT TGA GAG G	ACC TTG GAA AGT ACT GAG CG
NKX6.1	TGC TTC TTC CTC CAC TTG GTC CG	TCG TTT GGC CTA TTC GTT GG	TGT CTC CGA GTC CTG CTT C
PAX4	AGG AGT GGG AAG GAG ATG GCA TAG A	AAG GAC AAT GGG CAG GAT G	ACT GTA TGG CTT GGA ATG AGG
INS	CGG CGG GTC TTG GGT GTG TA	CTA GTG TGC GGG GAA CG	CAC GCT TCT GCA GGG AC
TBP	TGG GAT TAT ATT CGG CGT TTC GGG C	GAG AGT TCT GGG ATT GTA CCG	ATC CTC ATG ATT ACC GCA GC
GCG	ATG GCG CTT GTC CTC GTT CAT CT	ACG TTC CCT TCA AGA CAC AG	GTC CAG ATA CTT GCT GTA GTC AC
SST	AGG GCA TCA TTC TCC GTC TGG TTG	ACT CCG TCA GTT TCT GCA G	CTG GGA CAG ATC TTC AGG TTC

Dataset 1. Codeset details.

Available for download at
https://journals.biologists.com/dmm/article-lookup/doi/10.1242/dmm.052376#supplementary-data

Disease Models & Mechanisms • Supplementary information
